# Robust transmission of rate coding in the inhibitory Purkinje cell to cerebellar nuclei pathway in awake mice

**DOI:** 10.1371/journal.pcbi.1005578

**Published:** 2017-06-15

**Authors:** Samira Abbasi, Amber E. Hudson, Selva K. Maran, Ying Cao, Ataollah Abbasi, Detlef H. Heck, Dieter Jaeger

**Affiliations:** 1 Department of Biology, Emory University, Atlanta, GA, United States of America; 2 Department of Biomedical Engineering, Hamedan University of Technology, Hamedan, Iran; 3 Department of Anatomy and Neurobiology, University of Tennessee Health Science Center, Memphis, Tennessee, United States of America; 4 Computational Neuroscience Laboratory, Department of Biomedical Engineering, Faculty of Electrical Engineering, Sahand University of Technology, Tabriz, Iran; University College London, UNITED KINGDOM

## Abstract

Neural coding through inhibitory projection pathways remains poorly understood. We analyze the transmission properties of the Purkinje cell (PC) to cerebellar nucleus (CN) pathway in a modeling study using a data set recorded in awake mice containing respiratory rate modulation. We find that inhibitory transmission from tonically active PCs can transmit a behavioral rate code with high fidelity. We parameterized the required population code in PC activity and determined that 20% of PC inputs to a full compartmental CN neuron model need to be rate-comodulated for transmission of a rate code. Rate covariance in PC inputs also accounts for the high coefficient of variation in CN spike trains, while the balance between excitation and inhibition determines spike rate and local spike train variability. Overall, our modeling study can fully account for observed spike train properties of cerebellar output in awake mice, and strongly supports rate coding in the cerebellum.

## Author summary

Detailed computer simulations of biological neurons can make an important contribution to our understanding of how the brain works. In this paper we use such a model of a neuron that represents the output from the cerebellum. We can show that the inhibition this neuron type receives from Purkinje cells in the cerebellar cortex is well suited to pass a detailed time course of movement control to the output of the cerebellum. Importantly we find that this type of coding requires a population of Purkinje cells that pass the same temporal coding of spike rate to the output neurons in the cerebellar nuclei.

## Introduction

Transmission of information through firing rate changes in populations of connected neurons is one of the most widely accepted principles of neural coding. In motor control, for example, cortical neurons showing firing rate changes as a function of movement direction can be said to dynamically compute the current movement direction in a population vector [1]. This representation also works well computationally in abstract neural networks, for example when the motion of handwriting control is computed in the neural engineering framework [2]. Little is known, however, about how biological neurons utilize rate codes transmitted by their typically hundreds or thousands of input synapses to control their own output firing rate, and how robust such a code is in the presence of noise, intrinsic non-linearities given by voltage-gated channels, and a balance of excitatory and inhibitory inputs. Further, it is unclear whether rate codes are equally present in inhibitory as in excitatory transmission. We addressed these questions by studying the inhibitory transmission between cerebellar cortical Purkinje cells (PCs) and their targets in the cerebellar nuclei (CN) through recordings from awake mice and detailed biophysical simulations of synaptic integration in CN neurons. Linear rate coding has been identified to represent excitatory input information from granule cell input in PCs [3,4], but the correlation of coding at the population level and its transmission to CN neurons in vivo remains unclear. We use rhythmic motor patterns and in particular the rhythmic control of respiration as a model behavior to study the transmission of rate coding in cerebellar circuits, as rhythmic respiratory rate modulation is well expressed in the spiking activity of PCs in the cerebellar vermis [5] as well as in the synaptically connected medial (fastigial) cerebellar nucleus [6], and this pathway plays a functional role in the neural control of respiration [6].

In the present study we used an updated version of a full biophysical model of CN neurons [7] to study how the population of Purkinje cell inputs expected to converge on a single CN [8] may transmit a rate coded rhythmic behavior, and whether CN model generated spike trains can account for spiking properties recorded from CN neurons in awake mice. We developed a new algorithm that allows the flexible construction of sets of artificial PC spike trains that match the statistical properties of recorded PCs while also allowing the insertion of correlations observed between pairs of recorded PCs into a larger set of PC spike trains that converge onto a single CN neuron as input. This new algorithm development was necessary because it is at this time physiologically impossible to record from and identify all the PCs that converge onto a single CN neuron. Therefore, in order to simulate a realistic range of rate-correlations and respiratory coding correlations between the ~50 PC inputs received by a single CN neuron, it is necessary to generate populations of artificial spike trains (ASTs) in which each AST matches the statistics of PC recordings (which we obtained from awake mice) while flexibly allowing the addition of specific rate co-variances between ASTs. We achieved this goal by creating an intermediate representation of spike trains as rate templates that could be manipulated algebraically to show more or less rate co-variances both for respiratory related rate changes and slow rate fluctuations. To create ASTs we could then draw gamma distributed interspike intervals from the rate template to match the template’s rate fluctuations as well as the recorded spike train statistics. To our knowledge this study presents the first such algorithm, which we expect will be generally useful for similarly minded modeling studies of synaptic integration in the awake brain.

Our CN modeling results for the first time give a full match of CN spiking properties seen in awake recordings derived from the biophysical properties of CN neurons and the statistics of their synaptic inputs. The results reveal an unexpected amplification of rate coding at the CN output compared to the PC inputs received and show a highly robust transmission of rate codes from the cerebellar cortex to the CN via inhibition in the waking condition. They also provide evidence for an involvement of intrinsic cellular dynamics in providing gain control in the transmission of rate codes.

## Results

The starting point of our analysis was a database of 21 PC, 11 mossy fiber (MF) and 16 CN recordings. These data were obtained in awake head-fixed mice with multiwire recordings while respiration was monitored using a thermistor placed in front of one nostril [5,9]. Out of 20 PCs that were analyzed for rate modulation linked to respiration, 15 (75%) showed significant rhythmic rate modulation, as indicated by a deflection of the rate change in a peri-event time histogram (PSTH) triggered on inspiratory event markers above 3 standard deviations (S2 and S3 Figs). Standard deviations were calculated from a set of 100 control PSTHs from each cell that were calculated from randomly shifted spike time series with respect to the respiratory event markers. The same analysis showed significant respiratory modulation for 10 of 16 CN neurons (63%), and 6 of 9 (67%) MF recordings. This strong representation of respiratory activity supports previous evidence that the vermal cerebellar cortex through its output connection in the medial cerebellar nucleus is involved in the adaptive control of respiration [6]. The rate modulation for different cells showed different phase relationships to respiration, and the averaged rate modulation in the PC, CN, and MF neuron population was not significant (S3 Fig), suggesting that cerebellar respiratory modulation occurs at all phases of respiration to a similar degree, though in different populations of neurons.

We also made a detailed analysis of the recorded PC, MF and CN baseline spike train statistics, in particular firing rate as a function of time, interspike-interval (ISI) distribution, coefficient of variation (CV), and local variation (LV) [10], which indicates the variability of pairs of successive ISIs (S1 Fig, Table 1 in S1 Text). Our goal was to determine whether the spike train statistics and respiratory modulation of CN neurons can be explained from the dynamics of a biophysically realistic CN neuron model [7] and the input patterns received.

**Fig 1:**
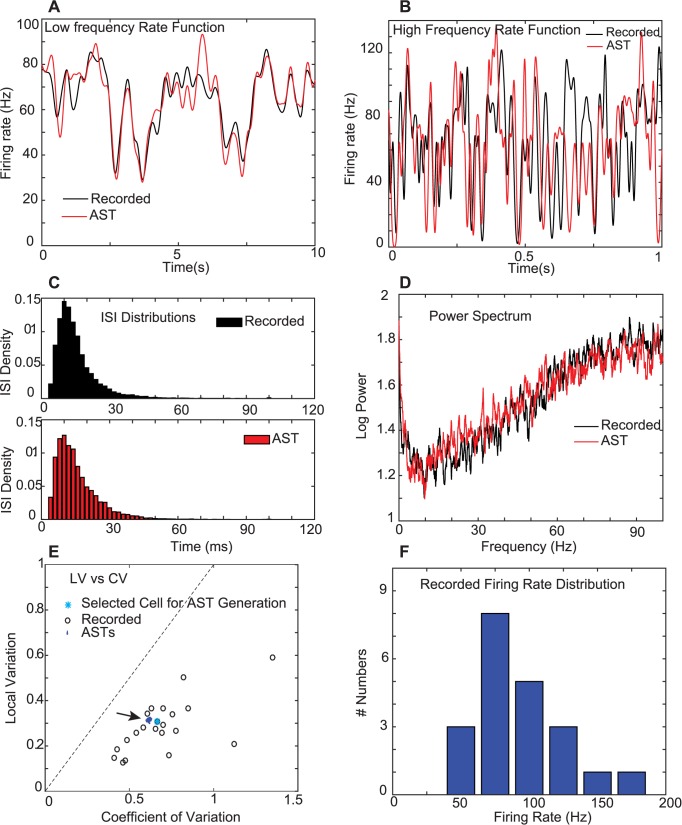
Purkinje cell AST construction from rate templates. Matching AST properties with recorded PC spike trains. **A.** The slow rate function calculated from a single recorded PC spike train (black) and of a sample AST sampled with a gamma distribution from the adaptive Gaussian rate function of this PC (see Methods). The AST generally matches slow rate modulations of the recorded PC. To highlight this match a representative time window of 10s out of the complete 115s spike train of the Purkinje cell recording was chosen. The mean difference between the recorded PC and AST rate functions over the complete 115s was 6.9 Hz (calculated as root mean square error–RMSE) **B.** The high frequency rate function was obtained by dividing the normalized adaptive rate function by the normalized slow rate function in order to isolate fast rate fluctuations. The AST matches the recorded fast fluctuations qualitatively, but not their exact time course. This is due to the random drawing of gamma ISIs in the creation of ASTs as fast fluctuations reflect this random process. To highlight this match for fast frequencies a representative time window of 1s out of the complete 115s spike train of the Purkinje cell recording was chosen. The time window shown corresponds to the 0-1s window in panel A. **C.** The ISI distribution of the 115s PC recording and of the refractory period corrected gamma AST are shown. The small mean difference in the ISI density of 0.0059 per bin (RMSE) indicates a good match between the recorded spike train and the AST derived from its rate function. **D.** The power spectrum of the same sample spike train shown in A-C (black) and of the AST constructed from the adaptive rate template (red). The match between 0 and 20 Hz is primarily due to the shared slow rate function and for faster frequencies is due to the fast rate function and to random gamma ISIs being matched to the shape parameter (LV) of the recorded spike train. The mean difference in Log Power across all frequencies was 0.072 (RMSE). **E.** The CV of recorded PC spike trains always exceeded the LV (dashed line marks unity) and exceeded the value of 1.0 for Poisson spike trains in some cases. ASTs (50 blue dots in a dense cluster) generated from a particular PC spike train (cyan asterisk) show the LV of the recording and a slightly diminished CV. **F.** The histogram shows the mean firing rate distribution of the 21 recorded PCs.

To achieve our goal we first had had to design a bootstrapping method by which to extrapolate from 2 simultaneously recorded PCs to a population of ~50 PC spike trains with flexible rate covariances that converge on a single CN neuron with strong synapes [8]. We determined that our recorded PC spike trains had broad cross-correlations that were in part related to behavior [9], but did not find any millisecond precision in simple spike cross-correlations here or in previous studies [11,12]. Complex spikes were removed from the PC spike trains, and not further considered in this study. We constructed a Matlab (MathWorks, Inc.) algorithm by which we can assemble artificial spike trains (AST) closely matching properties of single recorded PCs (Fig 1). The core of this algorithm consists of building and manipulating spike rate templates (Fig 1A and 1B), which are constructed by convolving spikes recorded from a single neuron with Gaussians [13,14] (see Methods). To construct ASTs we draw gamma distributed ISIs from a distribution with a mean rate tracking a rate template, and a shape parameter kappa (κ) that is mathematically derived from the LV of the recorded spike train, where for gamma distributed events LV = 3 / (2 κ +1) [10]. To obtain an ISI distribution in an AST that matches the original recording (Fig 1C) we further had to perform a refractory period correction, as gamma distributions do not model processes with refractory periods directly (see Supplemental Methods for details). We validated our ASTs by comparing the spike train power spectrum between recorded neurons and the built-to-match ASTs (Fig 1D), and by ascertaining that the coefficient of variation (CV) and the LV of the AST also matched the recording closely (Fig 1E). An important observation was that the LV could be modeled as a static parameter as previously observed for cortical neurons [15], but the global variability of the spike train over time represented by the CV is an outcome measure that is influenced by the LV as well as the spike rate modulation over time. Using these methods we made populations of 50 PC ASTs with statistical properties and spike rate fluctuations matching our recorded PCs while also being able to flexibly control rate covariances. All 50 PC ASTs used as input to the CN model were taken from the same master rate template of a single PC with specific different manipulations of rate-covariances for different simulation runs as described below (also see Supplemental Methods for details). These AST populations were then used to analyze how convergent input from 50 PCs would influence CN spiking, and what properties of convergent input were needed to account for observed CN spike train statistics.

**Fig 2:**
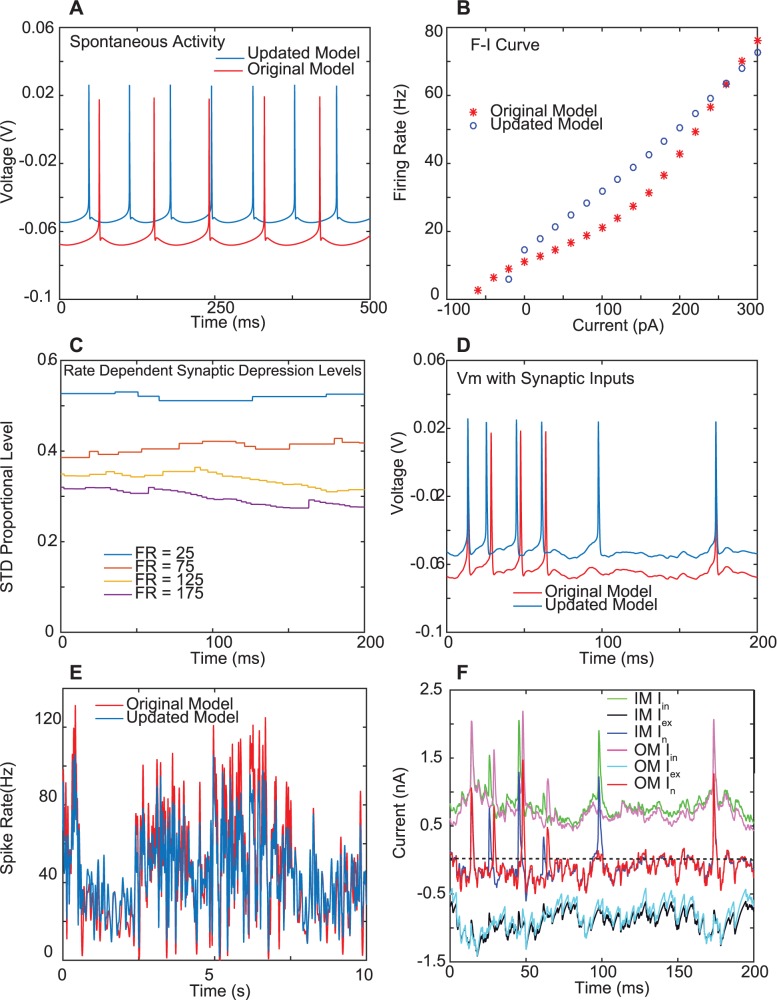
Properties of biophysical CN model. **A.** Spontaneous spiking in the absence of synaptic input. The original model is as published on ModelDB (https://senselab.med.yale.edu/modeldb/ShowModel.cshtml?model=136175), while the updated model includes the changes described in the Supplemental Materials of the original publication [7]. **B.** The model spike rate as a function of injected current. The rate was calculated as the inverse of the mean ISI over 1 s of injected current following 1 s of spike rate equilibration with the same current level. **C.** Synaptic depression levels in updated model during a sample period of 200 ms. The fraction of maximal synaptic conductance that is present in the absence of depression is shown for a sample PC to CN synapse activated randomly at the rates shown after steady state depression levels are reached. Each incremental change in depression is the result of the length of the preceding ISI in the PC input. (compare to Fig 2B in [20]). **D.** Vm trace comparison of original and updated model when subjected to the same mixed pattern of excitatory and inhibitory input (50 PC ASTs w/ G_in_ of 6 nS and 48 MF ASTs w/ G_ex_ of 2 nS. The GABA reversal potential of the original model is -80 mV, which is 10 mV more hyperpolarized than in the updated model). The spike times of both models similarly tied to fluctuations in inhibition and excitation. **E**. The spike rate of both models for the same set of input ASTs is similarly modulated. **F.** Synaptic currents in the model for the segment of activity shown in D). Note that the net (In) current, i.e. the sum of inhibitory and excitatory current is inward. Spikes in the inhibitory current are due to large driving force shifts during an action potential in the soma.

The biophysical CN model we used consists of 485 dendritic and one somatic compartments incorporating 9 active conductances to replicate slice CN recordings [7]. We included the modifications of ion channel voltage-dependence and density as well as synaptic kinetics described in the supplemental materials of the original publication ([7], S3 & S4 Figs), which lead to a more depolarized level of tonic depolarization (Fig 2A) and a more linear f-I curve (Fig 2B) as well as faster synaptic kinetics to more closely replicate CN slice recordings in these qualities [16,17,18,19]. In the present study we further modified the synaptic kinetics of PC->CN synapses to incorporate the experimentally determined short term depression parameters [20,21] leading to a steady state depression of around 60% for a Purkinje cell firing rate of 75 Hz (Fig 2C). The resulting spiking pattern with random excitatory and inhibitory input trains of the modified model remain similar to the original publication (Fig 2D and 2E), and are based on a balance of excitatory and inhibitory input currents with a fluctuating total synaptic current near zero (Fig 2F), which modulates the spontaneous activity of these neurons [16,22].

Next we characterized the CN model spiking output statistics for input patterns aimed to match the PC spike train statistics derived from our recorded data. We used 50 PC ASTs (48 dendritic, 2 on the soma) to match the number of strong PC inputs to converge on a CN neuron recently described [8]. We also applied 48 dendritic mossy fiber ASTs to create the required balance between excitation and inhibition [16,22]. We scanned through an array of input parameter settings that are not fully experimentally constrained, notably the size of unitary excitatory and inhibitory conductances (G_in_ and G_ex_), and the amount of rate covariances present between 50 synchronous PC inputs. The latter setting was manipulated by a shift fraction (SF), that is the proportion of rate modulation that utilized a randomly time shifted version of the master rate template. For the first set of simulations we used the PC firing rate of the template neuron (64.9 Hz) for all 50 ASTs. MF inputs to the model were also taken from a typical single recorded MF rate template, but as this study focused on the effect of rate covariances present in the PC pathway to influence CN spiking statistics we chose to use a SF of 1.0 for our baseline simulation (all MF inputs are temporally decorrelated) and an MF input rate of 20.4 Hz, which is the recorded sample mean.

**Fig 3:**
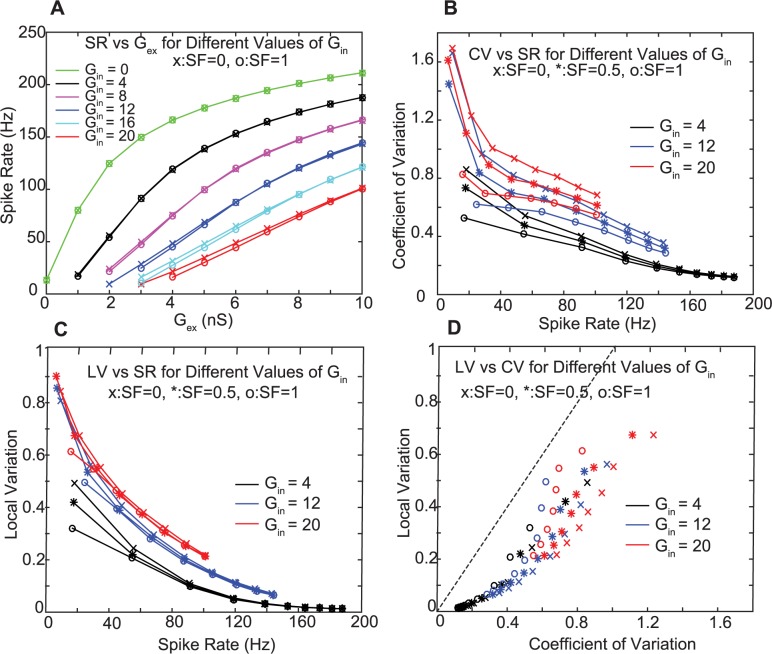
Model spike train statistics with PC and MF AST input matching recordings. **A.** Model spike rates for different gain factors in the unitary inhibitory input conductance (G_in_) as a function of unitary excitatory input conductance (G_ex_). There is little effect of 100% (SF = 0), and 0% (SF = 1) rate covariance between PC inputs on spike rate (symbols are superposed in many cases). **B.** The model spike train CV as a function of spike rate (SR) for different values of G_in_ and SF. Note that the CV for a given SR increases with increasing G_in_ and with decreasing SF. **C.** The LV also decreases with SR and decreasing G_in_. It is much less dependent on SF than CV because it is not sensitive to slow rate changes that result from rate covariance in the inputs. **D.** LV and CV are highly correlated, but the LV of model spiking is always lower than CV (dashed black line denotes unity).

**Fig 4:**
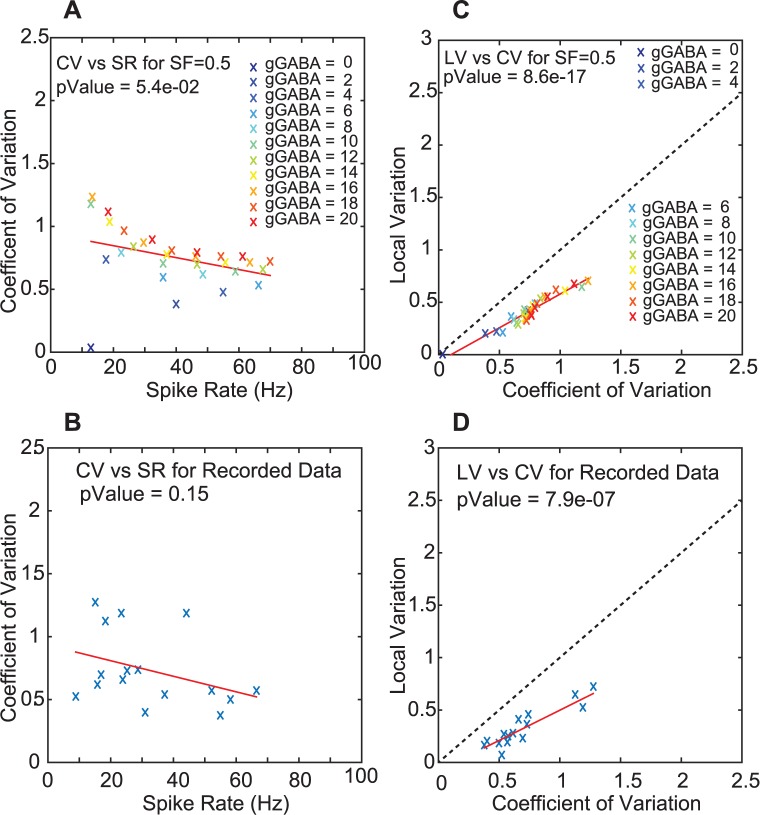
The CN model neuron spike statistics using different G_in_ and G_ex_ combinations fit the distribution of recorded spike train statistics. **A.** Model CV significantly decreases with SR (p-value for linear regression is given). Variability at any given SR is introduced by different values of G_in_ / G_ex_ such that higher total conductances result in a higher CV. **B.** The recorded CN neurons show a similar CV vs SR relationship but the CV variability at any given SR is higher. Each cross presents the mean SR of one recorded neuron. **C,D.** Model and data show a strong linear correlation between LV and CV. The recorded population is best matched by models employing inputs with a SF of 0.5 (subset of Fig 3D).

The results of this input parameter scan show that using different ratios of G_in_ and G_ex_ allowed us to achieve a wide range of CN output firing rates (Fig 3A), and revealed a systematic relationship between firing rates, CV and LV (Fig 3B and 3C) such that faster CN spike trains associated with a smaller G_in_ / G_ex_ ratio showed a lower CV and LV despite using the same PC input spike trains. Further, the CV and LV of CN spike trains were higher for larger absolute values of G_in_ (Fig 3B and 3C, red traces). For a high G_in_ (20 nS per PC input) the PC input rate covariance also had a strong effect on the CN output CV, such that a higher input rate covariance (Fig 3B, red traces with x symbols) resulted in a higher CN spike train CV. In contrast, the LV of CN spiking was much less affected by the input rate covariances (Fig 3C). A key result of our study is given by the match of the dependencies between CV and LV of CN spike trains between our simulations (Fig 4A and 4C) and our recorded CN data sample (Fig 4B and 4D) for the full range of physiological spike rates between 10 and 70 Hz. This simulation result indicates that the statistics of the PC and MF input to the CN as derived from our PC and MF recordings can fully account for the CN spike train statistics recorded in the same state. Interestingly, the match between recordings and simulation was best for a simulated SF of 0.5, indicating that the spike train statistics in the CN recordings are most compatible with PC input that contains about 50% rate covariance. Further, the variability between our CN recordings can be explained by a possible variability in total PC input conductance amplitudes received by different CN neurons and different rate covariances between these inputs. These results for the first time fully account for spike train statistics in the awake state in a biophysically based neural simulation. While certainly other factors than the PC input statistics can influence CN spike statistics in the animal, our results demonstrate that the PC input statistics alone are sufficient to account for the full spectrum of recorded CN rates and LV as well as CV statistics and their interdependence.

**Fig 5:**
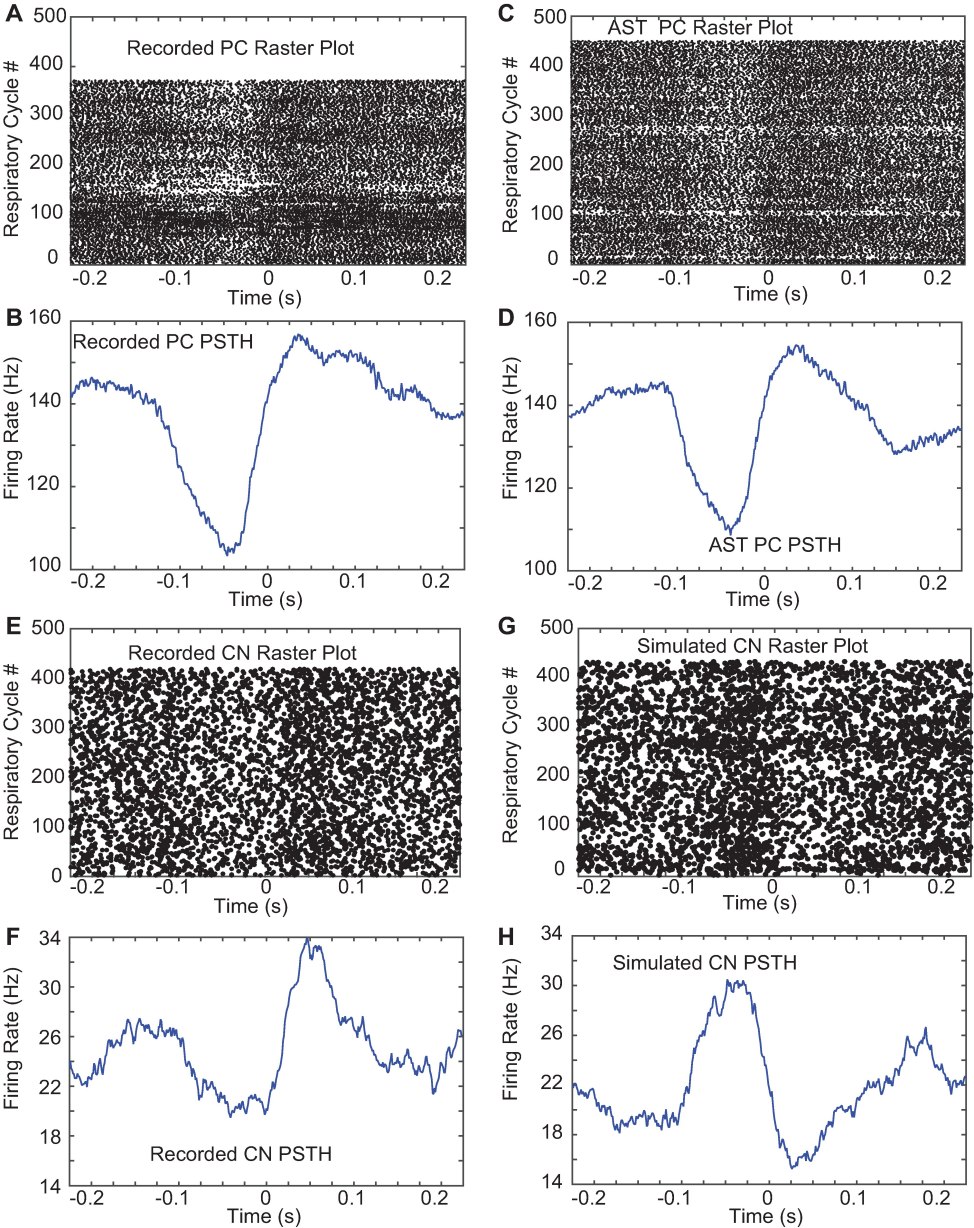
Recorded and simulated peri-stimulus time histograms (PSTH) for respiration. The median respiratory interval in our data set was 242 ms (respiratory frequency of 3.9 Hz), therefore approximately one full respiratory cycle is shown to each side of the event trigger. **A,B.** Spike raster plot for sample PC recording (top) and average PSTH (bottom). **C,D.** Spike raster plot for PC AST made from the shown sample average PSTH (A) convolved into the rate template from a different recorded PC without respiratory modulation at the time of each respiratory event. **E,F.** Raster plot (top) and average PSTH (bottom) of a sample CN neuron aligned to respiration. Note that this CN neuron is not recorded at the same time and its phase of modulation is not driven by the PC neuron shown in panel A,B. **G,H.** Simulated CN neuron respiratory PSTH resulting from simulation with 50% of PC inputs incorporating respiratory modulation as depicted in C,D). The dot sizes in the raster plots were adapted to the mean rate of each spike train to best depict modulation. Note that the phase of the modulation in the CN simulation is not targeted to match the phase of the CN recording, but is the inverse of the phase of respiratory modulation in the PC ASTs (Fig 5D) due to the inhibitory nature of PC inputs onto CN neurons.

Next, we aimed to incorporate the respiration related rhythmic spike rate modulation in the PC input to CN neurons in our simulations to determine whether the recorded PC respiratory modulation (Fig 5A and 5B) can explain the recorded CN modulation (Fig 5E and 5F). An important question not addressed by our recordings concerns the required level of covariance in respiratory modulation between PC inputs to a single CN to allow for the observed amplitude of CN respiratory modulation if it was solely transmitted by PC inputs. In order to create ASTs with respiratory modulation matching the recordings we again employed rate template manipulations. We determined the average rate modulation triggered by respiration in a given PC (Figs 5A, 5B, S2 and S3), and then we convolved the normalized rate modulation waveform with our master rate template at the measured time of each inspiration. We find that by drawing random gamma spike trains with refractory periods from this combined rate template we are able to create PC ASTs with respiratory rate modulation closely matching the experimental data (Fig 5C and 5D) while maintaining the spike train statistics of recorded PCs including their rate, LV, CV and power spectrum. As a proof of concept simulation we picked a specific CN recording with a peak of 36% spike rate increase during respiration (Fig 5E and 5F) and for our simulation input picked a G_in_ of 16 nS and G_ex_ of 3.5 nS, which we knew from our parameter scan to result in a matching mean baseline CN simulation spike rate of ~22 Hz. We then asked the question of how many of the 50 PC inputs need to show the respiratory modulation shown in our ‘typical’ PC recording (Figs 5A, 5B, S2A–S2C and S3A–S3C) in order to generate the behavioral modulation strength seen in our ‘typical’ CN recording (Figs 5E, 5F, S2D–S2F and S3D–S3F). The results showed that a respiratory modulation in 25 PC inputs (i.e. 50% of inputs) resulted in a match with our recorded CN modulation (Fig 5E–5H).

**Fig 6:**
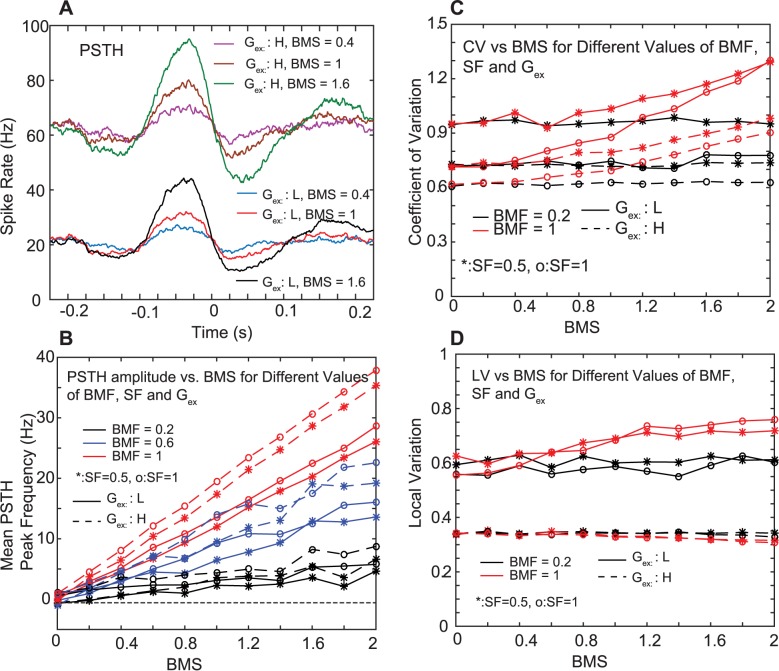
Model PSTH as a function of input AST properties. **A.** The average respiratory modulation in the spike output smoothly varies with the amplitude of respiratory modulation in the inputs (behavioral modulation strength (BMS): gain factor between 0 and 2 applied to the respiratory rate modulation of the sample recording from which ASTs were generated (Fig 5A and 5B)). The G_in_ was 16 nS, and the model was tested for 2 spike rates achieved with a high level of 6 nS and a low level of 3.5 nS for G_ex_. **B.** The amplitude of respiratory spike train modulation was scored as the mean frequency increase during the peak. The output spike frequency increase is nearly linear with increasing BMS of the inputs. The slope of this line is a function of the fraction of modulated inputs (BMF). The SF in the background rate covariance has little effect on the PSTH, but the frequency increases are higher when the baseline spike rate is 60 Hz than when it is 20 Hz. **C,D.** The CV and LV as a function of BMS for two values of BMF and 2 levels of excitation G_ex_: H(igh) resulting in a 60 Hz baseline, and G_ex_: L(ow) resulting in a 20 Hz baseline (see A). The CV is moderately affected by increasing BMS when the baseline spike rate is low.

Next, we determined the robustness and relative expression strength in the transmission of respiratory rate modulation in the PC -> CN pathway by systematically varying the number of modulated PC inputs and the strength of modulation in each input for a slow and a fast spiking CN simulation resulting from 2 different levels of excitation (Fig 6). We find that a change in the PC respiratory modulation strength is transmitted faithfully to the CN, and that respiratory modulation is well transmitted by slow or fast firing CN neurons (Fig 6A). Both the fraction of modulated PC inputs (BMF) and the strength of PC respiratory modulation (BMS) had strong effects on CN modulation (Fig 6B). At the strength of PC modulation present in our experimental sample PC (Fig 5A), a modulation of 10 of 50 PC inputs (BMF = 0.2) to the CN neuron was sufficient to result in a significant output modulation. If all PC inputs to the CN simulation were modulated using the 11.4% mean rate decrease in the PSTH trough observed in the sample PC, the CN mean PSTH peak rate increase was 25.9% at a firing frequency of 60 Hz, and 48.3% at a firing rate of 20 Hz, indicating that the respiratory modulation depth is amplified in the transmission from the PC to the CN in an inhibitory transmission. Strong respiratory modulation in the CN lead to a moderate increase in the CV of the CN spike trains (Fig 6C), while the LV was less affected (Fig 6D, G_ex_ = L. red solid lines). We further examined the effect of global rate covariances between PC inputs on respiratory modulation (SF 1.0 vs. 0.5), and found that this manipulation of background rate covariance only had a small effect on the transmission of respiratory modulation (Fig 6B, circle vs asterisk symbols), while it had a strong effect on the overall CV of the spike train (Fig 6C). As detailed in the Supplemental Information we found that the transmission of respiratory modulation was also robust against different PC input firing rates, the presence of absence of short term depression in the PC-> CN synapses, using rate templates from a different PC, and changing the gain on template rate fluctuations (S2–S7 Figs). The key outcome of these sets of simulations was that the inhibitory PC inputs on tonically active CN neurons provide a sensitive and accurate means of transmitting a rate code related to controlling behavior, and that the strength of this rate transmission is highly dependent on the fraction of inputs modulated with the same time course. Further, the transmission of rate modulation is robust in the face of common background rate modulation in the input and operates well for the full range of observed CN firing rates (10–70 Hz). Importantly our simulations demonstrate that the observed respiratory modulation in CN neurons can be fully explained by the measured rate modulation in PCs if at least 20% convergence of similarly modulated PCs onto single CNs is present.

**Fig 7:**
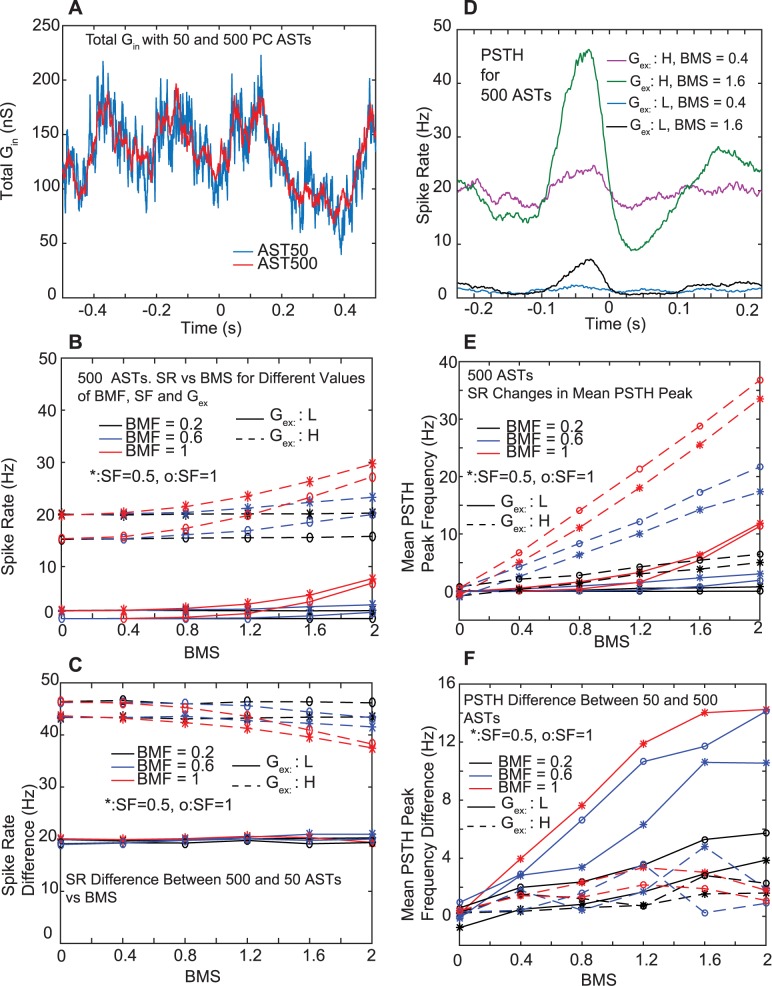
Comparison of CN simulations with 500 vs. 50 PC AST inputs. **A.** The unitary conductance was divided by 10 for 500 inputs to result in a matching mean inhibitory input conductance. **B,D,E.** Conventions as in Fig 6. **C.** The 500 PC inputs lead to a much reduced spike rate. **F.** Difference between (E) and Fig 6B. PSTH mean peak frequency changes are generally similar, but higher by up to 14 Hz for G_ex_: L with 50 PC inputs.

Earlier anatomical estimates of the number of PC inputs on CN neurons [23] were much higher than given by the recent physiological assessment [8]. While the recent work can account for the larger number of boutons anatomically observed by positing multiple boutons per PC input to a CN neuron, we were interested to know what the consequence of using 500 instead of 50 inputs would be for matching our recorded CN data from awake mice. We created 500 ASTs using the same rate template as previously for 50, but we divided the unitary synaptic conductance by 10 to arrive at a similar average conductance waveform (Fig 7A). A notable difference in the total conductance of 500 inputs was that high frequency fluctuations were much diminished due to averaging over 500 instead of 50 random processes. Notably, this had a large effect on the output spike rate from the CN simulation (Fig 7B and 7C), which was diminished for 500 inputs from 63 Hz to 20 Hz for a high level of excitation, and from 20 Hz to near zero for a low level of excitation. This dramatic difference illustrates the high importance for fast input conductance fluctuations in triggering individual sodium action potentials, a property not seen in integrate and fire neurons. We have previously also observed this finding in dynamic clamp experiments of CN neurons in brain slices [16]. Despite the large decrease in CN spike rate for 500 PC inputs, the respiratory modulation remained strong (Fig 7D–7F), and the absolute values of respiratory spike rate increases were nearly the same for a 20 Hz spike rate with 500 PC inputs than they were with a 60 Hz spike rate with 50 PC inputs with the same spike train properties (Fig 7E and 7F G_ex_: high, dashed lines). These findings again show that inhibitory synaptic transmission is a highly robust carrier for a behavioral event related rate code. The main computational outcome of using 500 instead of 50 PC inputs was that much more excitatory input is needed in order to match the spike rates in the model with those recorded in awake mice.

**Fig 8:**
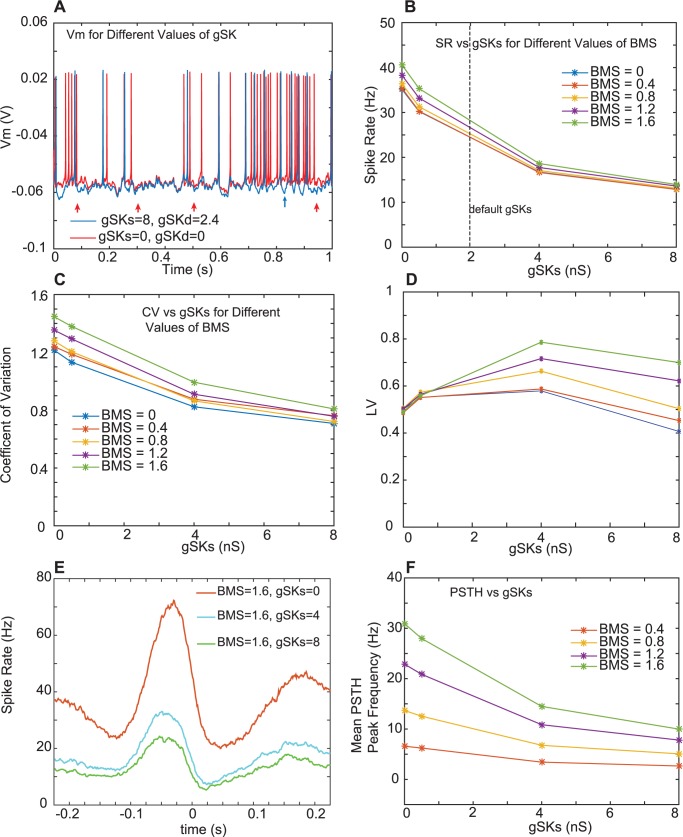
Effect of SK conductance density on model output. **A.** Sample period of spiking for zero (red trace) and 8 nS (blue trace) somatic gSk. Red arrows point to respiratory event times. Blue arrow points to a sample spike afterhyperpolarization (AHP), showing increased AHP depth for high gSK. **B.** Spike rate for identical AST inputs diminishes with increasing gSK. The behavioral modulation strength (BMS) has a minor effect on SR. **C.** The CV diminishes for increasing gSK. **D.** The LV shows a maximum for a gSKs around 4 nS. For a high gSKs the BMS has a noticeable effect on LV. **E, F.** The PSTH peak is diminished for higher values of gSKs.

Finally, we asked the question whether the intrinsic active currents of CN neurons make an important contribution to the spiking statistics and respiratory modulation in our simulations. While a full treatment of this question falls outside the scope of this study, we used manipulations of the density of the calcium dependent potassium current (SK) to see what contributions this modulatory current makes to CN coding properties in awake mice. In previous work we and others have shown that this current is present in CN neurons and that blocking it with apamin causes bursting, bistability and pronounced spike rate increases with depolarization [18,24,25]. Further, stochastic excitatory and inhibitory input patterns in the CN model lead to strong fluctuations in SK current [26]. The involvement of this important modulatory current in synaptic integration in the awake animal remains unknown, however. Our default simulation made to match typical CN slice recordings from 14-21d old rats had a somatic SK density of 2 S / m^2^ and a dendritic density of 0.6 S / m^2^. We varied these densities for SK densities between 0 and 8 S / m^2^ in the soma and proportionally 0 to 2.4 S / m^2^ in the dendrites (Fig 8). When SK was absent, comparing simulation Vm traces for 0 vs 8 nS SK density with the same synaptic input, we find a much reduced spike-afterhyperpolarization (example indicated by blue arrow in Fig 8A) and much stronger spike rate modulation for a given input rate modulation (Fig 8A, see 0.7 to 0.9s for a period of decreased inhibitory conductance), as should be expected from the biophysical properties of this potassium current that is activated via the calcium inflow with each action potential. Not surprisingly, there is also a systematic decrease in overall spike rate with increasing SK density (Fig 8B) and a decrease in CV (Fig 8C). The LV on the other hand shows a non-monotonic dependency on gSK, with a maximum near 4 nS (Fig 8D). While SK is known to regularize spike trains [27],this usually refers to the CV. The low LV when SK is absent is probably due to the high local regularity during periods of high frequency firing, but we did not further examine this effect. The effect of SK density on respiratory rate change transmission was also strong (Fig 8E and 8F). With increasing gSK the CN output PSTH rate modulation with the same input was much diminished. This result indicates that SK is well suited to dampen the transmission of behaviorally related rate changes. Interestingly SK appears to be downregulated in adult rodents [25], suggesting that as the cerebellum matures the gain of rate change transmission may be increased. Overall the strong effect of SK on spiking statistics and synaptic transmission of rate changes shows the high importance of intrinsic neuronal properties on the transmission of behaviorally related rate codes.

## Discussion

Our study posed the general question on how rate codes can be transmitted by inhibitory synaptic inputs using the cerebellar cortex to cerebellar nuclei projection as a paradigmatic example. This inhibitory connection is particularly interesting in that it conveys the entire output from the cerebellar cortex, and the cerebellar cortex is commonly thought to be involved in coding detailed temporal aspects of motor behavior [28,29,30]. Therefore, detailed temporal information has to be transmitted through the inhibitory cerebellar cortico-nuclear pathway. However, cerebellar research has generated conflicting ideas on whether this information is transmitted by a rate code [3,4] or by a temporal code triggered by input synchronicity [8,31]. While the distinction between rate and temporal codes can be blurry at intermediate values of temporal precision, one would generally take neural algorithms depending on coincidence detection [32], synfire chains [33] or input synchrony to detect patterns [34] as examples of a temporal code, while spike rate modulation at the time scale of the behavior controlled (which could be quite fast for saccades for example) represents a rate code. Our findings with respect to the coding of respiration in mice in this study are fully supportive of the rate coding model in the control of cerebellar output, as rate was smoothly varying on the time scale of the behavior observed. Our findings substantiate the concept that inhibitory synaptic transmission can convey such information with high accuracy in tonically active neurons. Nevertheless, it is entirely possible that a temporal code is multiplexed with this rate code, and would be triggered by specific events, such as motor errors. In the cerebellum such an event in particular is likely to be coded by highly synchronous climbing fiber firing [3,4], which could result in rebound activity in the CN [7,35,36,37]. This pathway should be analyzed carefully in future modeling work, but an experimental database of simultaneous cerebellar cortical and nuclei recordings in behaving animals while assessing climbing fiber synchrony is not yet available. For simple spike activity in cerebellar cortex in our mouse preparation we previously described an absence of synchronized spiking or synchronized pauses with respect to respiration and licking [5], or sensory activation in anesthetized rats [12]. Our modeling results in the present study show that indeed such coincident PC simple spike inputs to a CN neuron are not required to explain the observed rate, regularity or respiratory modulation of our CN recordings. Instead, we found that rate coding of PCs is fully sufficient to account for observed CN spiking properties, but that a substantial correlation in the rate modulation between PCs projecting to the same CN neuron is required.

We used a detailed biophysical CN neuron model to perform our investigation, which allows us to address the question of how much intrinsic active properties of CN neurons are important in decoding synaptic input. Interestingly, in the present input scenario of a time-varying balance of excitatory and inhibitory input the strong rebound firing capabilities of the model, which match experimental findings [7], did not come into play as significant de-inactivation of the rebound currents (T-type Calcium and persistent Na conductances) through strong hyperpolarization did not occur. Nevertheless, our investigation of the role of the SK conductance in the present study shows that the neurons’ active properties are highly significant in decoding synaptic input. Specifically, the SK conductance in CN neurons is known to cause prolonged spike-afterhyperpolarizations and regularize spontaneous spiking [17,24], which after block of SK current with apamin becomes highly bursty [17,24]. In a previous dynamic clamp study we showed that bursting is suppressed with a baseline of inhibitory and excitatory input conductance, but that the gain of responses to input modulation was increased when SK was low [25]. This role of SK controlling the gain of the synaptic response function was confirmed in our present modeling study for input conditions of respiratory spike rate modulation in the awake mouse. Recordings from slices of rodents at different ages suggest that SK is downregulated as animals become adult [25], thus perhaps allowing a greater CN output modulation by input fluctuations as the cerebellum learns to code for specific behaviors. However, even in the adult mouse the amount of SK current observed in single CN neurons may be highly variable as is typically observed for voltage-gated currents [38], and possibly serve as a gain control mechanism on the synaptic coding function of behavioral spike rate modulation that could be regulated through intrinsic plasticity. Such SK plasticity has not been studied in the CN, but is known to occur in other cell types [39].

While our results support the notion that modulated PC input on CN neurons is sufficient to explain observed CN spike train statistics and respiratory modulation, we do not wish to imply that mossy fiber inputs to the CN are irrelevant or ineffective in this regard. In our previous dynamic clamp studies in CN brain slice recordings we have shown that PC input alone can control CN spike rate and regularity in the presence of tonic excitation, which is required to achieve a necessary balance between excitation and inhibition [16]. However, when the MF input is also modulated in the dynamic clamp input to mimic in vivo input conductances [22], the MF activity can also control CN spiking, and that MF and PC triggered modulation of CN spiking is roughly additive. Nevertheless, we found that due to the high proportion of slow NMDA conductance in MF input to CN neurons [40,41] that CN spike train irregularity is predominantly caused by PC input transients [22]. Our current results lead to the prediction that the contribution of MF input to respiratory rate modulation would critically depend on the amount of respiratory rate-covariance in the MF inputs to a CN neuron. MF respiratory modulation, similar to PC modulation, shows a variety of phase relationships to respiration (S2 and S3 Figs), and therefore a mechanism to strengthen MF convergence with similar modulation on single CNs would be required. A detailed exploration of the required MF input parameters in order to be effective is outside of the scope of the present study, but will be undertaken in the future. Another implication that we do not wish to be taken from our modeling study is that the respiratory modulation transmitted from the PC to the CN does in fact control respiration. In fact, our working hypothesis is that baseline respiration is not controlled by the cerebellum, but that the observed coordination of different orofacial rhythms such as licking, swallowing, whisking with respiration [9] is effected through the connection of the medial cerebellar nucleus to the respective rhythm generators [42]. Establishing the functional role of cerebellar output on the coordination of these rhythm generators and the ability of the cerebellum to delay or advance the respiratory cycle when needed will require new experimental studies where these rhythms are challenged and the output of the cerebellum is optogenetically manipulated.

Any given neuron in the brain typically receives synaptic input from hundreds of other neurons. For the synaptic transmission of a rate code it is therefore critically important to understand what number of these inputs need to be rate co-varying, in order for a robust transmission of behaviorally related information. This point is closely related to the question of how population coding is instantiated in the brain, as enough neurons need to be participating in the same coding process so that their convergent connections on a target population would transmit a rate code accurately. To our knowledge this study is the first that quantifies the answers to these questions in the framework of a biophysically accurate model to match a data set recorded in awake animals. We find that in the cerebellum where 50 PCs converge onto a single CN neuron, transmission of significant rate modulation required about ~10 (20%) rate covarying PC inputs, while ~25 (50%) PC inputs resulted in an outcome matching one of the stronger CN rate modulation amplitudes found in our experimental data. This code was found to be robust against interference from both correlated and uncorrelated background noise. These modeling results make a strong experimental prediction that populations of PC neurons converging to single CN neurons need to show a larger shared behavioral rate modulation than is present in a random sample of single recorded PCs. While such data are not yet available, advances in calcium imaging at single cell resolution in combination with transsynaptic retrograde labeling may allow verification of this prediction in the near future.

We undertook a careful effort to characterize the global and local spike train statistics through assessing CV, LV, and power spectra. A considerable theoretical literature has been devoted to the significance of neuronal variability and its use to determine the statistical properties of spike trains and their functional relevance [10,43,44,45,46]. In particular, the presence of CV values greater than 1.0 that is characteristic of random Poisson processes has piqued the interest of theorists, and such values were present in some of our recordings. Previous work has related such high variability to spike initiation non-linearities [46,47] or dendritic coincidence detection [46,48], because an integrator over many random inputs would result in a very high degree of regularity in the output. However, our study suggests an alternative mechanism, by which high CV values result from rate covariances in the population of PC inputs to the model neuron. These input rate-covariances lead to constantly changing firing rates in the output as well, which increases the CV. A hallmark of this effect is that local spike train variability of 2 successive ISIs (LV) is much less affected and the outcome values of LV are smaller than the CV, unlike in random processes. Therefore, our data and simulations indicate that the assumption of a stationary statistical process underlying neuronal spike trains should be abandoned for the awake condition. Our method of using firing rate templates with specific proportions of co-variance to drive output spiking indeed capture the observed spiking irregularity of data from awake animals well. Parameterizing the degree of these covariances needed to match recorded spike train statistics allowed us to estimate of the required population rate covariance in the behaving animal, and such modeling can therefore shed some light on potential population coding properties in the brain. Another extensive line of theoretical and modeling work has focused on ‘balanced state’ networks, where inhibition and excitation are matched [44,49,50,51]. These recurrent networks of integrate and fire neurons can show Poisson irregularity in firing [50], and a CV > 1 when co-varying sensory rate fluctuations are transmitted [51]. The required balance between excitation and inhibition in this network state is similar to the balance of excitation and inhibition needed in synaptic input applied to CN neurons with dynamic clamping in order to result in irregular firing patterns with random input spike trains [16,22], a property well replicated in our model [26]. The present study extends this work to the awake state and demonstrates that these concepts fully suffice to explain the spike train statistics recorded in awake alert mice when rate covariance between inputs is added.

## Methods

### Experimental data set

Animals. Experiments were performed on male and female adult C57BL/6J (B6) mice (18–25 g; The Jackson Laboratory). All mice used in this study were raised and all experiments were performed in accordance with procedural guidelines approved by the University of Tennessee Health Science Center Animal Care and Use Committee under protocol # 13–077. Details about surgical procedures to implant a head post and a recording chamber over the cerebellum were previously published [5,52]. During recording mice were head-fixed to a metal holder and the body was loosely covered with a plastic tube to limit body movements. Respiratory behavior was monitored with a thermistor (Measurement Specialties) placed in front of one nostril. Breathing cycles were measured as increasing and decreasing temperature changes caused by exhale and inhale movements, respectively. Peaks and troughs in the respiratory signals corresponded to the ends of expiration and inspiration cycles, respectively. Trough times were detected from the analog thermistor output sampled at 1 KHz and used throughout this study as respiratory event markers for respiratory spike train modulation and as alignment for respiratory peri-event histograms.

Up to seven recording electrodes (glass-insulated tungsten/platinum; 80 μm O.D.; impedance, 3–7 MΩ) were inserted acutely into the cerebellum during each recording session using a computer-controlled microdrive (System Eckhorn; Thomas Recording). Vermal Purkinje cells were identified by recording depth, a high spontaneous activity rate, and the presence of complex spikes [53]. Mossy fibers were identified using previously described criteria based on granular layer identification and spiking characteristics [54]. Single unit recordings from CN neurons were identified by electrode depth, the electrode passing through an area without spiking activity (i.e. the white matter embedding the CNs) before reaching the nucleus, and finally by the presence of sustained spiking (~10–70 Hz) without the occurrence of complex spikes. Recording locations were verified by placing small electrolytic lesions during the last 2 recording days and anatomical reconstruction from 50 μm coronal sections with a cresyl violet staining to align lesion sites with stereotaxic atlas coordinates [55]. Spikes were sorted off-line using Spike2 software (Cambridge Electronic Design) and only neurons with a clear refractory period in the ISI histogram and stable spike size over at least 45 s were used for further analysis. This resulted in a data set of 21 PCs, 11 MF, and 16 CN neurons.

### Determination of respiratory rate modulation

Spike trains were aligned on respiratory event markers (end of inspiration) to create a respiratory PSTH. A confidence interval (z-score) to determine significant modulation was constructed by shuffling the respiratory event times 100 times and creating a shuffled PSTH for each instance. Respiratory modulation exceeding the 95% confidence percentile for multiple data points in sequence was deemed significant. The amplitude of modulation was scored by the area under the largest peak or trough of the modulation after baseline subtraction and was scaled to units in spikes, thus yielding a measure of the number of spikes adding or missing in the PSTH peak or trough compared to the shuffle predictor.

### Construction of PC and MF artificial spike trains matching awake recordings

Using Matlab (MathWorks, Inc.) we designed an algorithm to create artificial spike trains (AST) that could replicate the observed spike trains statistics and respiratory modulation. We could not directly use experimentally recorded spike trains to drive our CN simulation input because we had at most triple simultaneous PC recordings whereas 50 simultaneous spike trains are needed as input to the model. We used cross correlation and spike covariance analysis [5] to determine the types of cross-correlation and rate covariance present between pairs of simultaneous spike trains and designed an algorithm that could extrapolate these properties to larger spike train populations. Our algorithm uses as core concept the method of rate templates, which are constructed from recorded spike trains by convolving each spike with a Gaussian (see Supplemental Methods, the full Matlab algorithm is available on ModelDB). In the next step of the algorithm we drew gamma distributed spike trains using a mean ISI tracking our rate template and using the shape parameter κ (kappa) experimentally determined by the LV from our recordings (see Results). This method allowed us to add respiratory rhythmic modulation in spike trains in a flexible way, by convolving rate templates with a gain-scaled version of the mean peri-stimulus time histogram (PSTH) triggered by each cycle of respiration. In order to convolve rate templates and respiratory PSTH functions independent of absolute firing rates, both rate functions were normalized to 1.0 before combining them, and the resulting combined rate function was scaled back to the desired mean firing rate of the output AST. Our new method of creating ASTs is quite general and could be used to incorporate any other known rate changes related to behavior. We expect that this method will be of general use in the neural simulation community.

### CN neuron model specifications

In this study we utilized our existing 486 compartment model including the updates to the voltage dependent conductances and synapses described in the Supplemental Information of the original publication and previously shown to replicate CN firing with stochastic synaptic input patterns applied by dynamic clamping [26]. This model has a set of 6 voltage gated and 1 calcium dependent conductance to match the spike shape, spontaneous firing, and responses to depolarization/hyperpolarization of slice CN recordings closely. It also includes 2 inactivating inward conductances that control rebound bursting after strong hyperpolarization [7]. These rebound conductances were present in the model used here, but due to the lack of strong hyperpolarizations with the input patterns constructed to match the waking condition they remained largely inactivated and rebound firing was not observed. In the present study, we included one further model update by incorporating a detailed version of the short term plasticity rules in the PC synapses on CN neurons experimentally determined [20,21]. The model depression rule is based upon the rate dependent release probabilities at multiple release sites as estimated by Telgkamp and Raman, 2004. These STD rules required a re-write of the Genesis 2.3 synchan object base code as they could not be achieved with existing synaptic mechanisms in Genesis. The new C base code as well as the updated model definition are available in ModelDB, https://senselab.med.yale.edu/modeldb/ShowModel.cshtml?model=229279.

Synaptic inputs were modeled as a dual exponential alpha function with rise and decay time constants matching voltage clamp recordings in slices (see detailed explanation in supplemental materials, [7]). Each spike from our PC ASTs triggered a unitary IPSC with a peak amplitude controlled by the G_in_ parameter. The range of G_in_ used was between 2 and 20 nS for parameter scans, and values of 4 or 16 nS were used in most simulation runs exploring respiratory rate modulations. This compares to an average IPSC size of 9.4 nS with minimal (single axon) stimulation in slices and an observed experimental range of 1–25 nS. Our excitatory MF inputs triggered both an NMDA and an AMPA EPSC, as mixed currents have been found in experiments [40,41,56]. 48 inhibitory and 48 excitatory synapses were distributed randomly across the 485 dendritic compartments, and 2 inhibitory synapses were placed on the soma. There was no somatic excitation as excitatory synapses are not observed on the soma of CN neurons [57,58]. Each synapse was connected to one AST input spike train.

### Simulation runs and analysis

Simulations were run in batches on a Linux cluster, where each batch completed a matrix of parameter settings. All simulations were run to produce 115s of output data. Binary and spike event output files from simulation batches were put into a Pandora database format and analyzed with custom made Matlab scripts. Power spectra were determined using functions from the Chronux Matlab toolbox (http://chronux.org/).

## Supporting information

S1 Text Supplemental information text. List of Abbreviations, Supplemental Methods, Supplemental Results, Supplemental Tables, and Supplemental References.
(PDF)

S1 Fig Spike train statistics of recorded neurons. A. Histogram of mean spike rates for all 21 recorded PCs. The recording durations per neuron ranged from 45.8–256.8s, with a mean of 116.9 s. B. The CV as a function of spike rate for all 21 recorded PCs. C. The LV as a function of spike rate for all 21 recorded PCs. D-F) Plots for 16 CN neuron recordings. Recording durations were between 600 and 256.8s with a mean of 118.2 s. G-I) Plots for all 11 MF recordings. Recording durations were between 40.1 and 120.0s with a mean of 71.1s.
(PDF)

S2 Fig Respiratory modulation of recorded neurons. A. Spike raster histogram centered on respiratory event times (end of inhalation as denoted by coldest thermistor reading) for a single PC. B. Spike rate of the same PC plotted for each row of the raster histogram and shows an average of 450 ms spiking. C. Peri-respiratory spike rate modulation for the spike data shown in A. The black trace shows the average spike rate modulation centered on the inhalation event times (PSTH). The blue trace shows an average of 100 control PSTHs, in which the spike time matrix was shifted to varying random degrees with respect to the inhalation event times. Shifted PSTHs were used in order to preserve the temporal statistics of spike rate changes as well as the temporal statistics of the respiratory event times. This method allows for the best estimate of the spike rate noise contribution to PSTH waveforms. The cyan traces show ± 2 standard deviations derived from the 100 shifted PSTHs. The red trace shows a single shuffled PSTH, in which a set of uniformly random event times of the same number as respiratory event makers throughout the recording period were used. This shuffled PSTH was deemed the best method to decorrelate event alignment times from spike rate changes related to respiration. Note that due to the regular nature of respiration, a time shifted version of the inhalation event markers or spike trains as used in our control PSTHs to estimate standard deviations may result in significant peaks of the shifted PSTH due to long periods of spurious alignments. All raw calculated PSTHs were binned with 1ms precision, and smoothed with a 100ms running average filter in order to dampen high frequency noise peaks. D-F) Respiratory modulation of a sample CN neuron. G-I). Respiratory modulation of a sample MF. While the selection of sample PSTHs in this figure used examples with similar phase relationships to respiration, this was not a constant property across recorded cells (see S3 Fig).
(PDF)

S3 Fig PSTH population properties. A. The PSTH of 5 PCs with significant respiratory modulation is shown. PSTHs here are normalized to their mean rate to indicate the proportional rate increases and decreases during respiration. The dark blue PSTH corresponds to the PC also shown in S1A–S1C Fig. All PSTHs here are smoothed with a 30ms running average. B. The PSTH peaks and troughs for all 21 analyzed PCs are shown. Each cell is represented with the maximal firing rate increase and decrease shown within a 225ms time window before and after the inspiration event time. Note the large spread of peak times. C. The average of all 21 PC normalized PSTHs is shown. D-F) Same analysis for CN neurons (N = 16). The cyan colored PSTH corresponds to the recording highlighted in S1D–S1F Fig. CNs also show a wide distribution of phases in rate changes locked to respiration, and a flat summed PSTH. G-I) Same analysis for MF recordings (N = 9).
(PDF)

S4 Fig The influence of short term depression (STD) in the PC to CN pathway. Conventions as in Fig 6. Differences in panel (B,D) are between STD-off v. on. Negative values denote that the values for STD on simulations were smaller. The panels matching (A,C) for STN-on are shown in Fig 6.
(PDF)

S5 Fig The influence of halving the PC AST spike frequency. The Gin was adjusted from 16 to 27.52 nS to result in the same level of total inhibition as for the default PC spike rate (64.86 Hz = population mean of recordings). The adjustment was less than double because the steady state depression level at half the firing rate was reduced. Panel annotation and methods used are as in Figs 6–8. For panels showing differences (D,F) the outcome is compared to the default simulation (Fig 6). Negative numbers denote an increase over the default.
(PDF)

S6 Fig The influence of doubling the PC spike rate. The Gin was adjusted from 16 to 9.84 nS to result in the same level of total inhibition as for the default PC spike rate. The adjustment was less than half because the steady state depression level at double the firing rate was increased. Panel annotation and methods used are as in Figs 6–8 and S5.
(PDF)

S7 Fig The influence of using a natural distribution of PC input spike rates. The Gin was set to 12.37nS for this set, as this resulted in a good match for the total inhibitory input conductance. Different PC spike rates for ASTs were obtained from the same rate template by scaling the normalized template to a distribution of rates across ASTs matching the recorded rate distribution. This gives a higher weight to faster spiking inputs than lower spiking ones, which is partly offset by the different levels of steady state depression, however. Panel annotation and methods used are as in Figs 6–8 and S5.
(PDF)

S8 Fig The influence of using PC inputs with a different rate template. The PC with the new rate template had a spike rate of 59.9 Hz, a lower CV of 0.43 (compared to CV = 0.67 for the default), and a lower LV of 0.18 compared to LV = 0.31 for default). Panel annotation and methods used are as in Figs 6–8, S4 and S5.
(PDF)

S9 Fig Simulation outcomes for different amplitudes of slow and fast rate modulations in the PC master rate template. Gex = 3.5 nS, Gin = 16 nS, BMS = 0.8, BMF = 0.8 for all panels.
(PDF)
