# Supplementary material for: Robust transmission of rate coding in the inhibitory Purkinje cell to cerebellar nuclei pathway in awake mice
Source: PLoS Comput Biol. 2017 Jun 15;13(6):e1005578. doi: 10.1371/journal.pcbi.1005578 (PMC5491311; doi:10.1371/journal.pcbi.1005578)
Supplement: S1 Text — List of Abbreviations, Supplemental Methods, Supplemental Results, Supplemental Tables, and Supplemental References. (PDF) [file pcbi.1005578.s001.pdf]

List of Abbreviations used in Paper and in Supplemental Materials

aGrf: Adaptive Gaussian rate function

BMS: Behavioral Modulation Strength

BMF: Behavioral Modulation Fraction

CN: Cerebellar Nuclei

CV: Coefficient of Variation

FRM: Fast Rate Modulation

LV: Local Variation

MF: Mossy Fiber

PC: Purkinje cell

RP: Refractory Period

SF: Shift Fraction

sGrf: slow Gaussian rate function

SR: Spike rate

SRM: Slow Rate Modulation

Supplemental Methods

Construction of PC and MF artificial spike trains matching awake recordings

Rate Template Manipulations: For a spike train of a given mean rate an optimal Gaussian width (denoted by σ (sigma)) exists that maximizes the information contained in a rate function constructed by convolving each spike time with this Gaussian (Paulin and Hoffman, 2001). For a given mean spike rate (SR) and a CV of 1.0 the optimal Gaussian σ to calculate a rate function has been determined to be 2 / (SQRT(2 π) *SR), but spike trains with a lower CV have a narrower optimal σ (Paulin and Hoffman, 2001). Because our firing rates were fluctuating at slow as well as at fast time scales we designed a 2 step adaptive Gaussian method by which we first convolved all spikes with a wide Gaussian (σ = (1 / (SQRT(2 π) *4s^{-1}) ≈ 100 ms) to create a slow Gaussian rate

function (sGrf) tracking slow spike rate changes (s) and then used this sGrf to determine a variable narrower Gaussian with $\sigma = 1 / (SQRT(2 \pi) * SR)$, where SR is time dependent and read from the sGrf. This created an 'adaptive' Gaussian rate function (aGrf), with a faster temporal resolution that was conditioned on slow spike rate changes. We normalized the sGrf and aGrf to a mean of 1.0 to allow important rate template manipulations to flexibly create AST populations with varying rate co-modulations. First, we could selectively gain-scale slow and fast rate fluctuations by making a new mixed rate template (mGrf) by multiplying the normalized sGrf (slow rate fluctuations) and (aGrf / sGrf) (fast rate fluctuations remaining after dividing out sGrf) with a gain factor and then multiplying the gain scaled slow and fast components back together. This manipulation was required to produce ASTs with different power spectra to match recorded PC spike trains with more or less pronounced low frequency power. However, it was only used in the production of Fig S9, and otherwise the aGrf was used. Second, we could flexibly control the amount of shared rate modulation in a population of ASTs by multiplying the aGrf with a randomly shifted version of itself (shiftedGrf) and weighting the shifted components with a shift fraction (SF), and weighting the unshifted version with 1-SF. For example an SF of 0.5 would introduce 50% rate co-modulation in the ASTs. Using the shift function rather than adding noise ensured that the statistical properties of biological firing rate changes remained intact. Third, we could convolve the aGrf with a normalized PSTH of behavioral modulation at the times of respiratory events that were recorded experimentally. This would introduce a respiratory modulation into the aGrf that resulted in ASTs matching the original respiratory modulation in a recorded PC (Fig. 5). Our master aGrf was taken from a PC without respiratory modulation so that we could add in any strength of behavioral modulation (BMS) into ASTs by convolving the master aGrf with gain-scaled respiratory PSTH functions. We could further introduce this behavioral modulation only into a specified fraction (BMF) of the AST population that drove our CN model.

<u>PC AST generation:</u> PC ASTs were constructed from gamma distributed inter-spike intervals, where each interval is drawn from a distribution with a mean μ and a shape parameter κ. The μ we used was time varying and read out from the aGrf (or mGrf) which was scaled from the normalized version to the desired mean spike rate. The κ

we used was constant across the AST, which allowed good fits with recordings and is agreement with data from cortex (Shinomoto et al., 2003). A previous theoretical study proved that κ is mathematically related to the LV of a gamma distributed spike train as LV = 3 / (2 κ +1) (Shinomoto et al., 2005). The LV used to determine κ was determined from recorded PC spike trains after subtracting the RP. While more complex than a previous published version of using gamma distributions to create ASTs (Shimokawa and Shinomoto, 2009) or other approaches (Brette, 2009; Macke et al., 2009), our method including refractory period corrections and rate templates from recordings resulted in better matches between recorded data and ASTs, and allowed us to control rate covariances between ASTs through rate template manipulations. We believe that this algorithm will be widely applicable beyond PC recordings with a caveat that firing rates near zero will need special treatment because gamma distributed ISIs drawn from near zero rates are so long that they will jump over rate changes in our slow rate function. This problem was largely avoided in our fast firing PC and MF recordings, but we did have to introduce a correction to our algorithm for some exceptionally low spike rate periods. To do so we introduced a floor of spike rate templates at 2 Hz, and we monitored the aGrf for sudden rate increases during a randomly drawn gamma ISI, and an additional spike was placed when the rate function jumped 4-fold during any given ISI. This resulted in a total fraction of up to 0.017 of all spikes being added this way when the BMS was set to 2, which provided an exacerbated case of rate modulation.

MF AST generation: Only one set of MF ASTs was generated, which was constructed from a recorded MF spike rate template and targeted the mean rate and regularity (LV) of the recorded MF population. The RP was also set to 3 ms. A shift fraction of 1.0 to decorrelate all MF inputs and a behavioral rate modulation of 0 were used to isolate the transmission of PC rate modulation to CN neurons.

Supplemental Results

Population coding in Purkinje cells.

An important question regarding population coding is whether a random connectivity between populations of neurons would transmit a viable rate code. This would only be the case if the average PSTH with respect of the rate modulation to be transmitted was significantly modulated. This is the case for example in some basal ganglia output signals, where movement initiation related signals are relatively uniform in phase across neurons and the averaged PSTH shows a strong signal (Mallet et al., 2016). When all respiratory related activity across PC (Fig. S3C), CN (Fig. S3F), or MF (Fig. S3I) recordings is averaged we find no significant rate modulation in the population PSTH, indicating that the different phase relationships of PSTH peaks and troughs exhibited by different cells cancel in the average. Therefore, a long term plasticity rule strengthening synapses with respiratory rate covariances is needed to transmit rate modulation from PC to CN levels, and likely also for the MF to PC and possibly the MF to CN pathway. Such plasticity rules could pick out distinct phase relationships for different connected populations.

Robustness of rate code transmission from cerebellar cortex to the CN via inhibitory PC output.

We performed multiple additional simulation batches testing specific aspects of robustness in the rate code transmission by varying parameter settings that were fixed in our results reported in the paper.

First we analyzed the influence of short term depression (STD) on spike statistics and the transmission of respiratory rate changes. We turned off STD and compensated for the lack of depression by decreasing the unitary G_{in} from 16 to 7.2 nS, which for the PC mean spike rate of 64.9 Hz used results in the same average level of total inhibitory input conductance. We find that the CV and LV are lower when STD is on than when it

is off when there is significant rate co-modulation between PC inputs (Fig. S4A-D). This makes sense as STD works to suppress the effects of input rate changes on output spiking by counteracting weight changes on the synapses. There are almost no differences in the expression of PSTH peak frequencies for STD on vs. off (Fig. S4E,F; cf. Fig. 6 B). We conclude that STD has little impact on PSTH transmission when the firing rate is uniform. The most likely function of STD would be a normalization of PSTH information carried by PCs with different firing rates that converge onto a single CN neuron (Chance et al., 2002).

Next we analyzed the influence of different PC spike rates on spike statistics and the transmission of respiratory rate changes. In one batch we halved the PC spike rate from 64.9 to 32.5 Hz (Fig. S5), in another batch we doubled the PC spike rate from 64.9 to 129.8 Hz (Fig. S6), in a third batch we used the recorded firing rate distribution (Fig. 1F) scaled to 50 ASTs as inputs (Fig. S7), and in 4th batch we used the rate template from a different PC (Fig. S8). For all simulations we adjusted the unitary G_{in} of PC inputs to the CN model so that the total average inhibitory input conductance had the same value. This was important to avoid strong effects of a shifted excitation/inhibition balance on spike rates, which would create a confounding factor in the analysis of PC rates on CN spiking. In vivo a similar scaling could occur through homeostatic plasticity mechanisms. Halving the PC spike rate lead to an increase in fast fluctuations in the total PC input conductance to the CN model (Fig. S5A), while doubling the frequency reduced these conductance fluctuations (Fig. S6A). This lead to about a 15 Hz CN spike rate increase for half the input frequency (Fig. S5C,D), and a decrease by 6-8 Hz for double the input frequency (Fig. S6C,D). This effect is due to the same reasons of changed input fluctuations following from smaller or greater number of total synaptic inputs per second as discussed for the 500 AST input condition described in the main results (Fig. 7). The transmission of respiratory rate modulation was nearly unchanged for both half and double PC input frequency, however (Fig. S5-S6. B,E,F), highlighting the robustness of this population level rate code. The results from a distributed PC input frequency (Fig. S7) were in all respects quite close to the default simulation of 64.9 Hz, and resulted in only minor decreases in CN spike rate (Fig. S7C,D) and minor fluctuations in the transmitted PSTH (Fig. S7 B,E,F). Finally, we used a rate template

from a PC with a similar spike rate (59.9 vs. 64.9Hz) but a noticeably lower CV (0.43 vs 0.67) and LV (0.18 vs 0.31) than the default PC used for all other AST constructions. The spike rates and PSTH peaks of the CN output remained nearly unchanged for inputs from this different PC (cf. Fig. 6A vs S8B, Fig. 6B vs S8C), indicating that our main simulation results were not dependent on the chosen rate template. However, the CV of the simulation output was about 0.1 higher with the default PC input when the shift fraction of the inputs was set at 0.5 (Fig. S8F, traces denoted with asterisks). This signifies that the lower overall rate modulation of this different PC as visible by its lower CV results also in a lower CV of the model output. This effect is much weaker for a shift fraction of 1, when the rate modulation is shifted among inputs, and the resulting CV is overall lower as already discussed for Fig. 6C in the main paper. The LV of the model output is also following the higher LV of the default inputs (Fig.S8H), but in this case the effect is strongest when the excitatory input conductance is low, i.e. the CN model spike rate is also low. This results signifies that more regular firing PC inputs lead to more regular spiking CN outputs, but this effect is washed out by generally regular CN spiking at high spike rates. A low shift fraction of PC inputs further decreases the lowered LV at low CN spike rates (Fig. S8H). Overall this set of simulations revealed a significant influence of the CV and LV of CN firing on the CV and LV of its PC inputs, and again a transmission of behavioral rate changes that is robust to such differences.

Lastly we determined the effect of a relative change in fast or slow rate modulation amplitudes in the PC ASTs. This was accomplished by multiplying the fast and slow rate functions (Fig. 1A, B) with a gain factor SRM (for slow rate modulation) or FRM (for fast rate modulation) before recombining them into the original adaptive Gaussian rate template (see Supplemental Methods above). All simulations performed before had FRM and SRM gain factors of 1.0 preserving the original rate modulation amplitudes present in the adaptive Gaussian representation of the recorded PC. The motivation for this parameter scan was that we observed PC recordings with power spectra showing different relative amplitudes in the slow and fast frequency bands, and we wanted to determine if such differences had a notable impact on CN output spiking. We find that the CN spike rate is increased for both an increased SRM as well as an increased FRM. For the sample case of (G_{in} = 16 nS, and G_{ex} = 3.5 nS) shown, the CN spike rate

increased by ~5 Hz for a change in SRM from 0 to 2, and by ~3 Hz for a change in FRM from 0 to 2 (Fig. S9A). The CV increased strongly from ~0.8 to ~1.6 for a change in SRM gain from 0 to 2 while the FRM had a smaller effect and a higher FRM lead to a lower CV (Fig. S9B). While we did not fully investigate these effects, the counterintuitive result of a lower CV resulting from a higher gain of FRM appears to be due to such FRM breaking up very long ISIs that appear at a low rate in the SRM, and which increase the CV significantly. Effects on LV were not pronounced but showed a small decrease with higher SRM gains and a small increase with higher FRM gains. As with previous parameter manipulations the transmission of respiratory modulation was hardly affected (Fig. S9C) and again proved remarkably robust.

Supplemental Tables

Table S1. Statistics of recorded spike trains

	Mean rate (Hz)<br><br>(SD / Min / Max)	CV<br><br>(SD / Min / Max)	LV<br><br>(SD / Min / Max)
PC (N=21)	92.1 (33.9 / 37.8 / 168.0)	0.69 (0.22 / 0.41 / 1.35)	0.29 (0.12 / 0.13 / 0.59)
MF (N=11)	20.4 (10.7 / 9.9 / 38.8)	0.86 (0.41 / 0.22 / 1.75)	0.34 (0.12 / 0.09 / 0.47)
CN (N=16)	32.5 (17.6 / 8.8 / 66.4)	0.73 (0.30 / 0.38 / 1.27)	0.34 (0.19 / 0.07 / 0.72)

Supplemental References

Brette R (2009) Generation of Correlated Spike Trains. Neural Comput 21:188-215.

Chance FS, Abbott LF, Reyes AD (2002) Gain modulation from background synaptic input. Neuron 35:773-782.

Macke JH, Berens P, Ecker AS, Tolias AS, Bethge M (2009) Generating Spike Trains with Specified Correlation Coefficients. Neural Comput 21:397-423.

Mallet N, Schmidt R, Leventhal D, Chen F, Amer N, Boraud T, Berke JD (2016) Arkypallidal Cells Send a Stop Signal to Striatum. Neuron.

Paulin MG, Hoffman LF (2001) Optimal firing rate estimation. Neural Networks 14:877-881.

Shimokawa T, Shinomoto S (2009) Estimating Instantaneous Irregularity of Neuronal Firing. Neural Comput 21:1931-1951.

Shinomoto S, Shima K, Tanji J (2003) Differences in spiking patterns among cortical neurons. Neural Comput 15:2823-2842.

Shinomoto S, Miura K, Koyama S (2005) A measure of local variation of inter-spike intervals. Biosystems 79:67-72.
