# Supplementary figures and images for: Robust transmission of rate coding in the inhibitory Purkinje cell to cerebellar nuclei pathway in awake mice
Source: PLoS Comput Biol. 2017 Jun 15;13(6):e1005578. doi: 10.1371/journal.pcbi.1005578 (PMC5491311; doi:10.1371/journal.pcbi.1005578)

Supplemental Figure 1

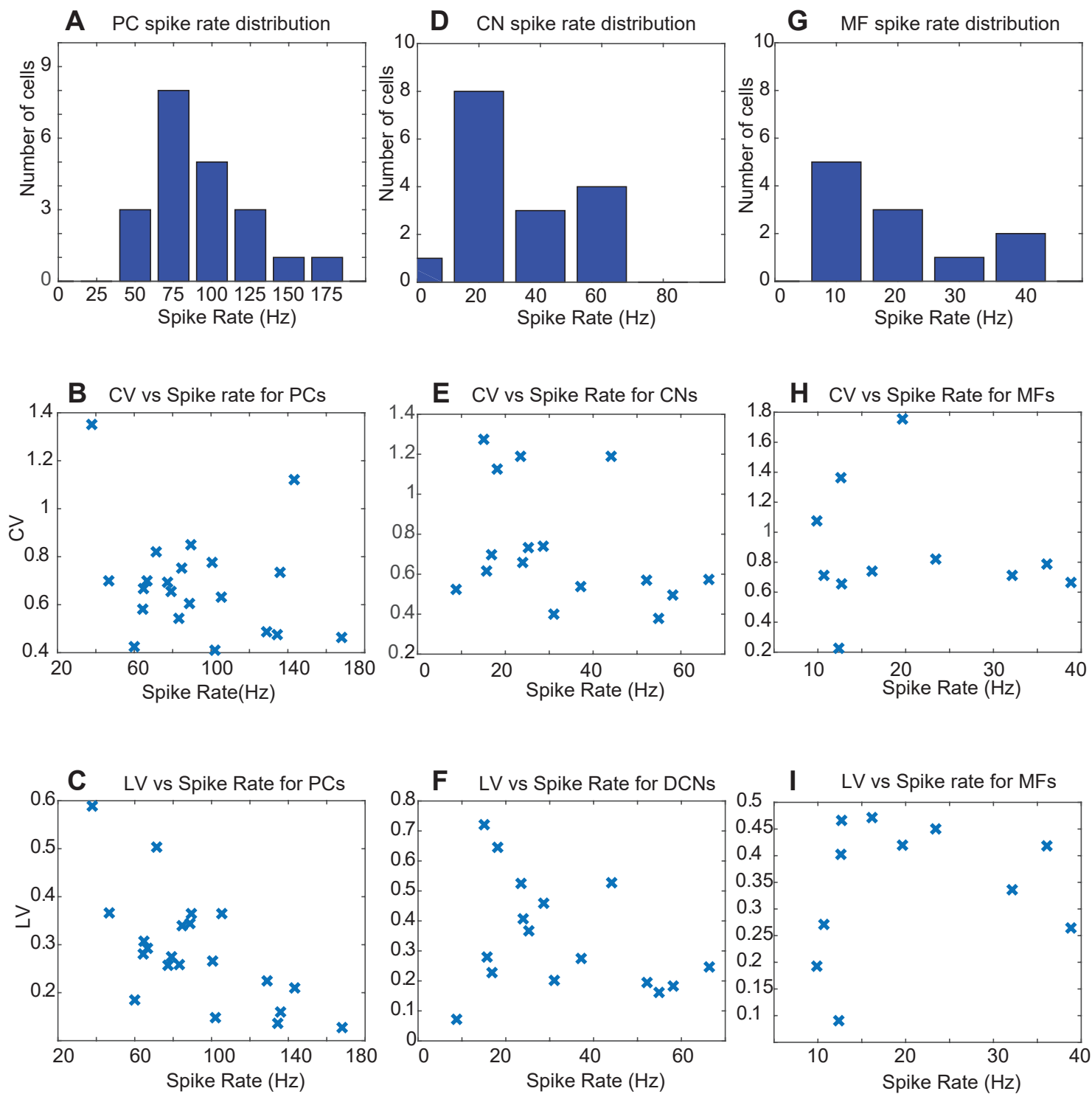

Supplement: S1 Fig — A. Histogram of mean spike rates for all 21 recorded PCs. The recording durations per neuron ranged from 45.8–256.8s, with a mean of 116.9 s. B. The CV as a function of spike rate for all 21 recorded PCs. C. The LV as a function of spike rate for all 21 recorded PCs. D-F) Plots for 16 CN neuron recordings. Recording durations were between 600 and 256.8s with a mean of 118.2 s. G-I) Plots for all 11 MF recordings. Recording durations were between 40.1 and 120.0s with a mean of 71.1s. (PDF) [file pcbi.1005578.s002.pdf]

Supplemental Figure 2

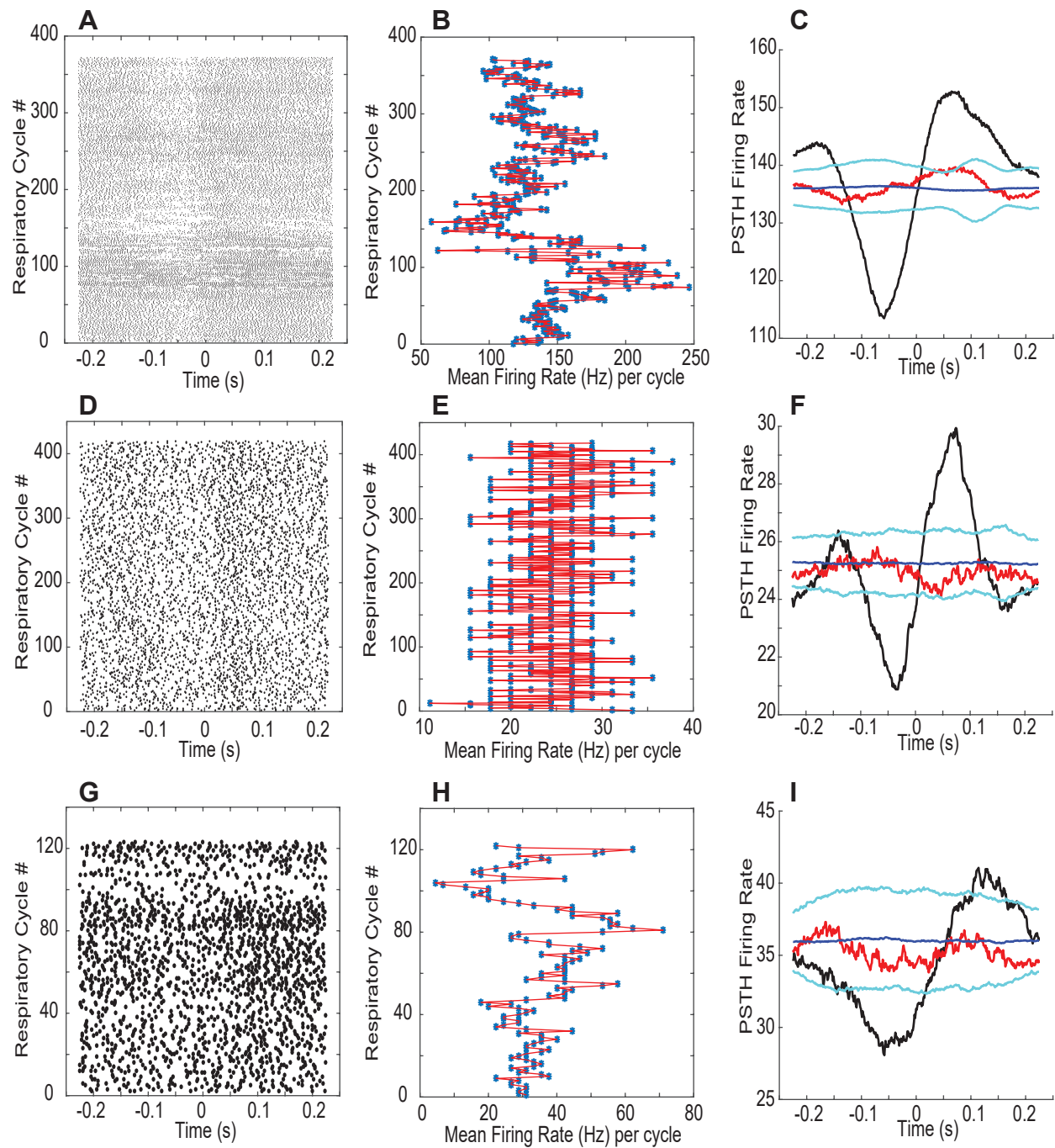

Supplement: S2 Fig — A. Spike raster histogram centered on respiratory event times (end of inhalation as denoted by coldest thermistor reading) for a single PC. B. Spike rate of the same PC plotted for each row of the raster histogram and shows an average of 450 ms spiking. C. Peri-respiratory spike rate modulation for the spike data shown in A. The black trace shows the average spike rate modulation centered on the inhalation event times (PSTH). The blue trace shows an average of 100 control PSTHs, in which the spike time matrix was shifted to varying random degrees with respect to the inhalation event times. Shifted PSTHs were used in order to preserve the temporal statistics of spike rate changes as well as the temporal statistics of the respiratory event times. This method allows for the best estimate of the spike rate noise contribution to PSTH waveforms. The cyan traces show ± 2 standard deviations derived from the 100 shifted PSTHs. The red trace shows a single shuffled PSTH, in which a set of uniformly random event times of the same number as respiratory event makers throughout the recording period were used. This shuffled PSTH was deemed the best method to decorrelate event alignment times from spike rate changes related to respiration. Note that due to the regular nature of respiration, a time shifted version of the inhalation event markers or spike trains as used in our control PSTHs to estimate standard deviations may result in significant peaks of the shifted PSTH due to long periods of spurious alignments. All raw calculated PSTHs were binned with 1ms precision, and smoothed with a 100ms running average filter in order to dampen high frequency noise peaks. D-F) Respiratory modulation of a sample CN neuron. G-I). Respiratory modulation of a sample MF. While the selection of sample PSTHs in this figure used examples with similar phase relationships to respiration, this was not a constant property across recorded cells (see S3 Fig). (PDF) [file pcbi.1005578.s003.pdf]

Supplemental Figure 3

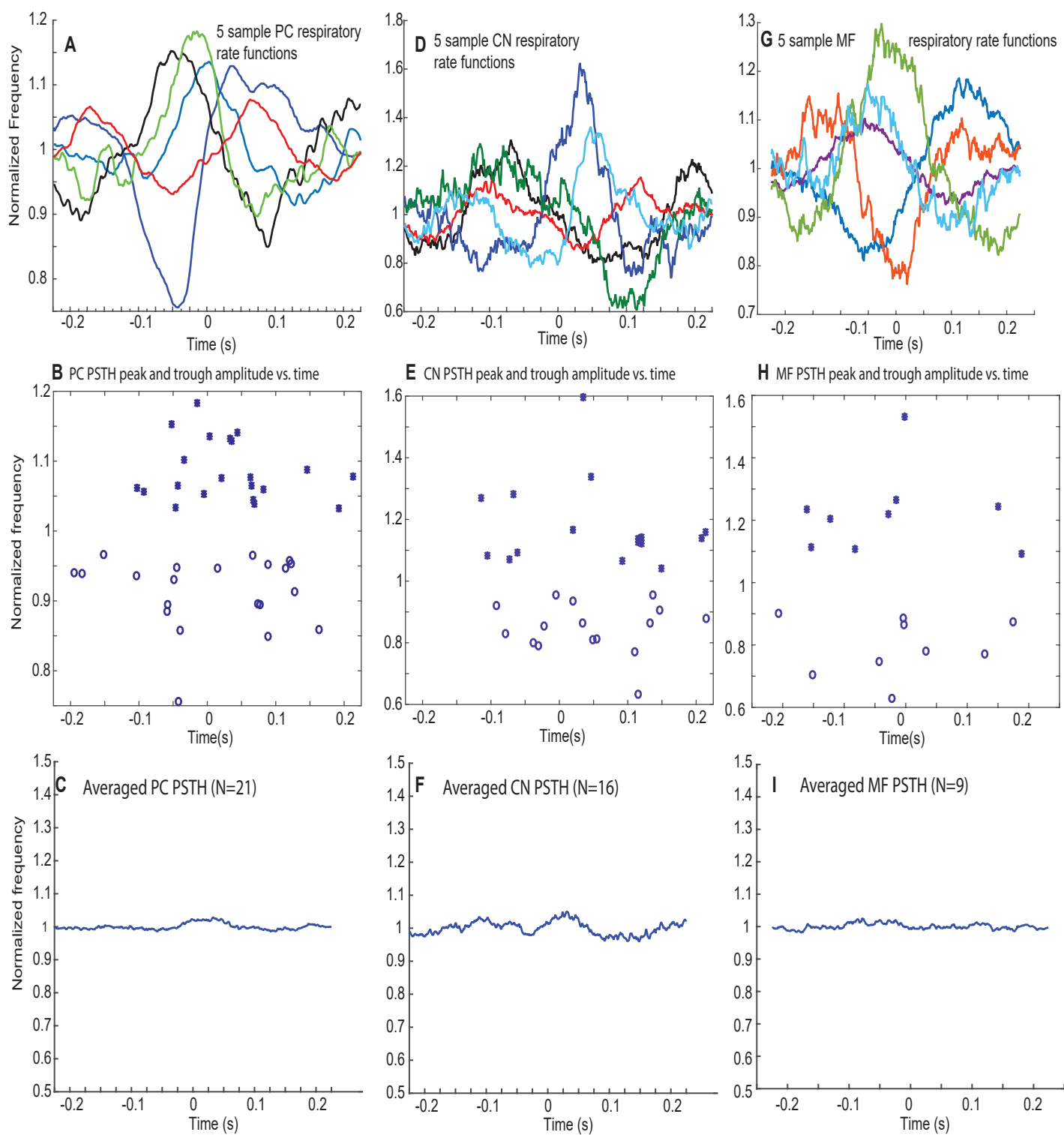

Supplement: S3 Fig — A. The PSTH of 5 PCs with significant respiratory modulation is shown. PSTHs here are normalized to their mean rate to indicate the proportional rate increases and decreases during respiration. The dark blue PSTH corresponds to the PC also shown in S1A–S1C Fig. All PSTHs here are smoothed with a 30ms running average. B. The PSTH peaks and troughs for all 21 analyzed PCs are shown. Each cell is represented with the maximal firing rate increase and decrease shown within a 225ms time window before and after the inspiration event time. Note the large spread of peak times. C. The average of all 21 PC normalized PSTHs is shown. D-F) Same analysis for CN neurons (N = 16). The cyan colored PSTH corresponds to the recording highlighted in S1D–S1F Fig. CNs also show a wide distribution of phases in rate changes locked to respiration, and a flat summed PSTH. G-I) Same analysis for MF recordings (N = 9). (PDF) [file pcbi.1005578.s004.pdf]

Supplemental Figure 4

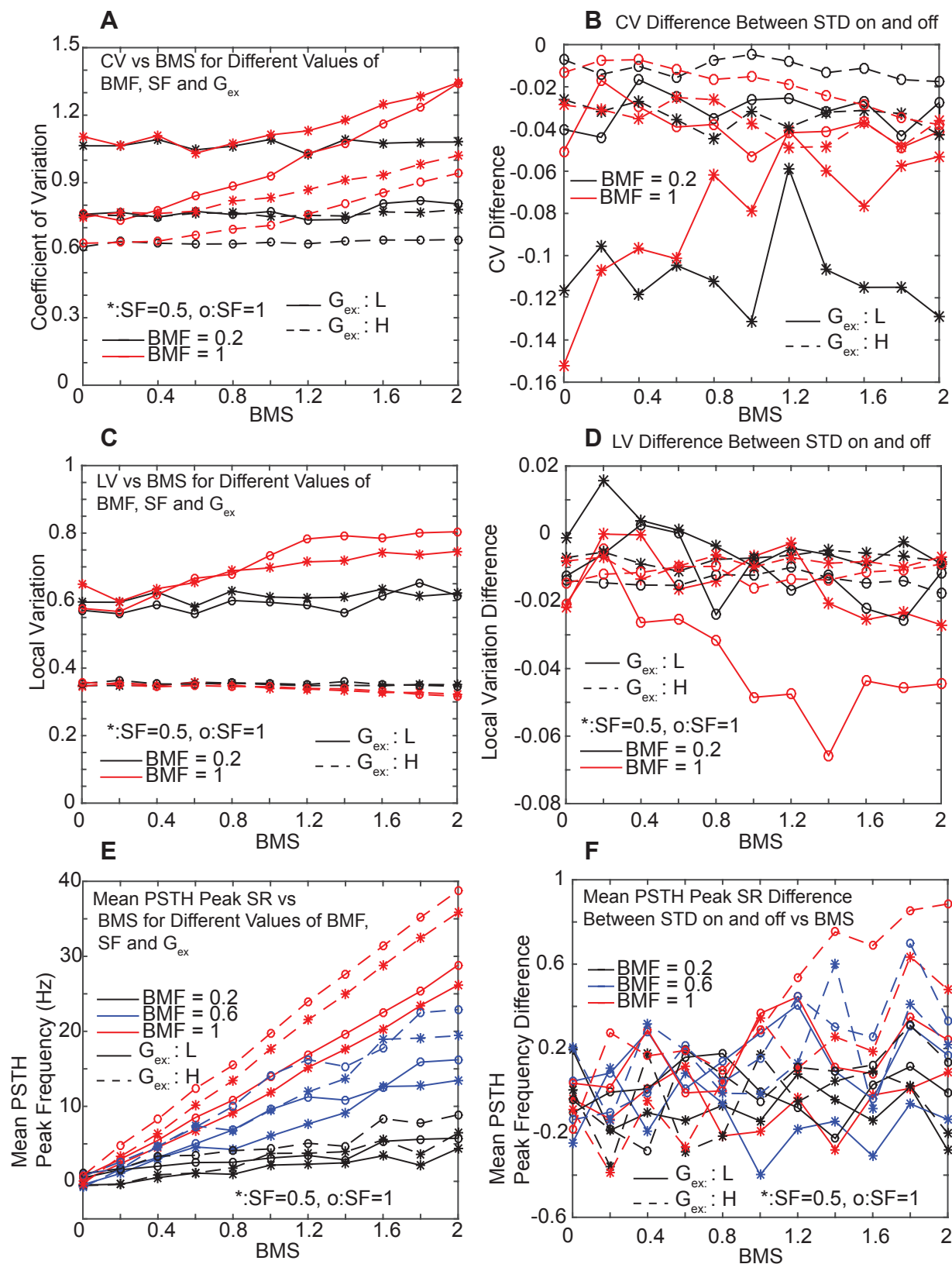

Supplement: S4 Fig — Conventions as in Fig 6. Differences in panel (B,D) are between STD-off v. on. Negative values denote that the values for STD on simulations were smaller. The panels matching (A,C) for STN-on are shown in Fig 6. (PDF) [file pcbi.1005578.s005.pdf]

Supplemental Figure 5

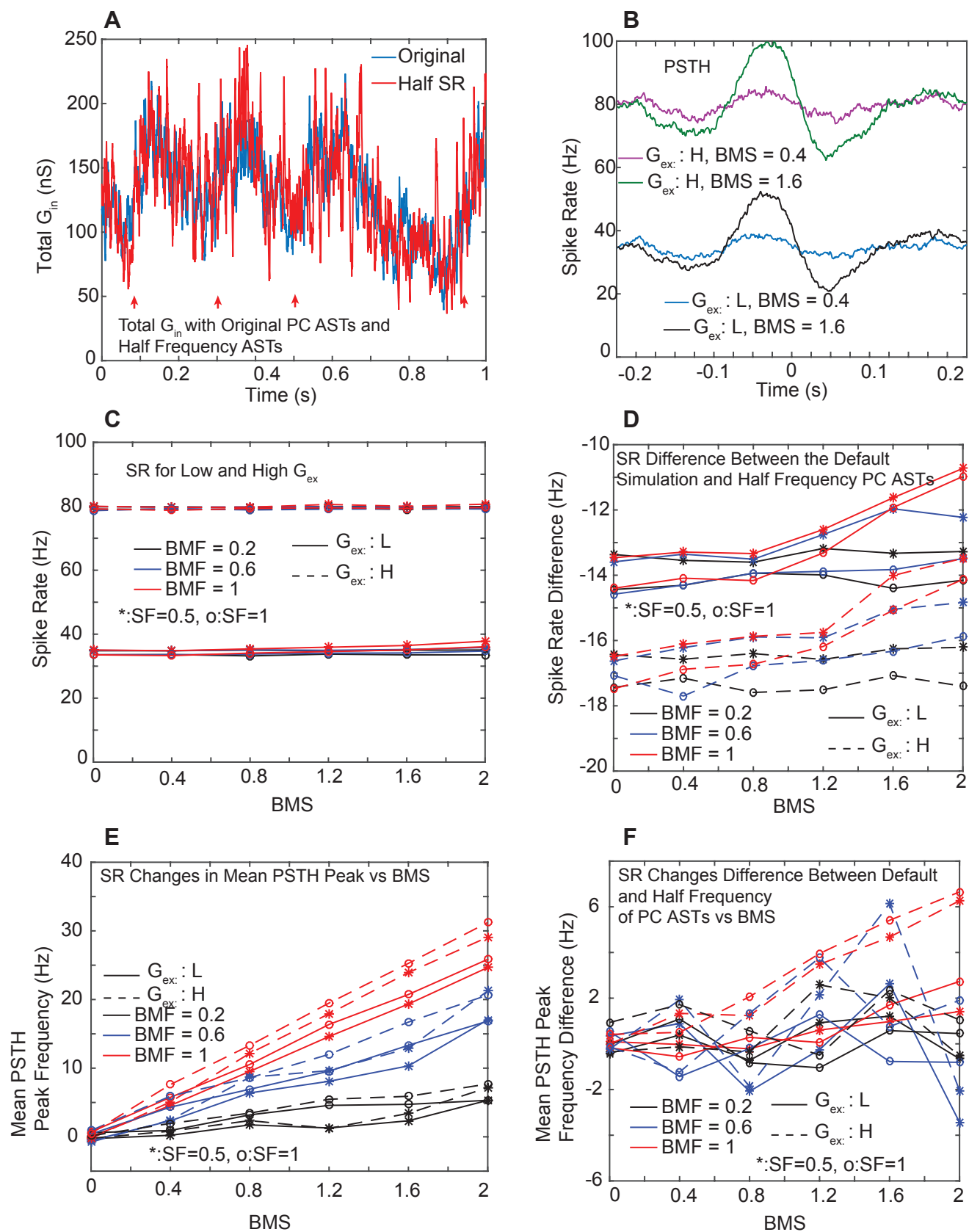

Supplement: S5 Fig — The Gin was adjusted from 16 to 27.52 nS to result in the same level of total inhibition as for the default PC spike rate (64.86 Hz = population mean of recordings). The adjustment was less than double because the steady state depression level at half the firing rate was reduced. Panel annotation and methods used are as in Figs 6–8. For panels showing differences (D,F) the outcome is compared to the default simulation (Fig 6). Negative numbers denote an increase over the default. (PDF) [file pcbi.1005578.s006.pdf]

Supplemental Figure 6

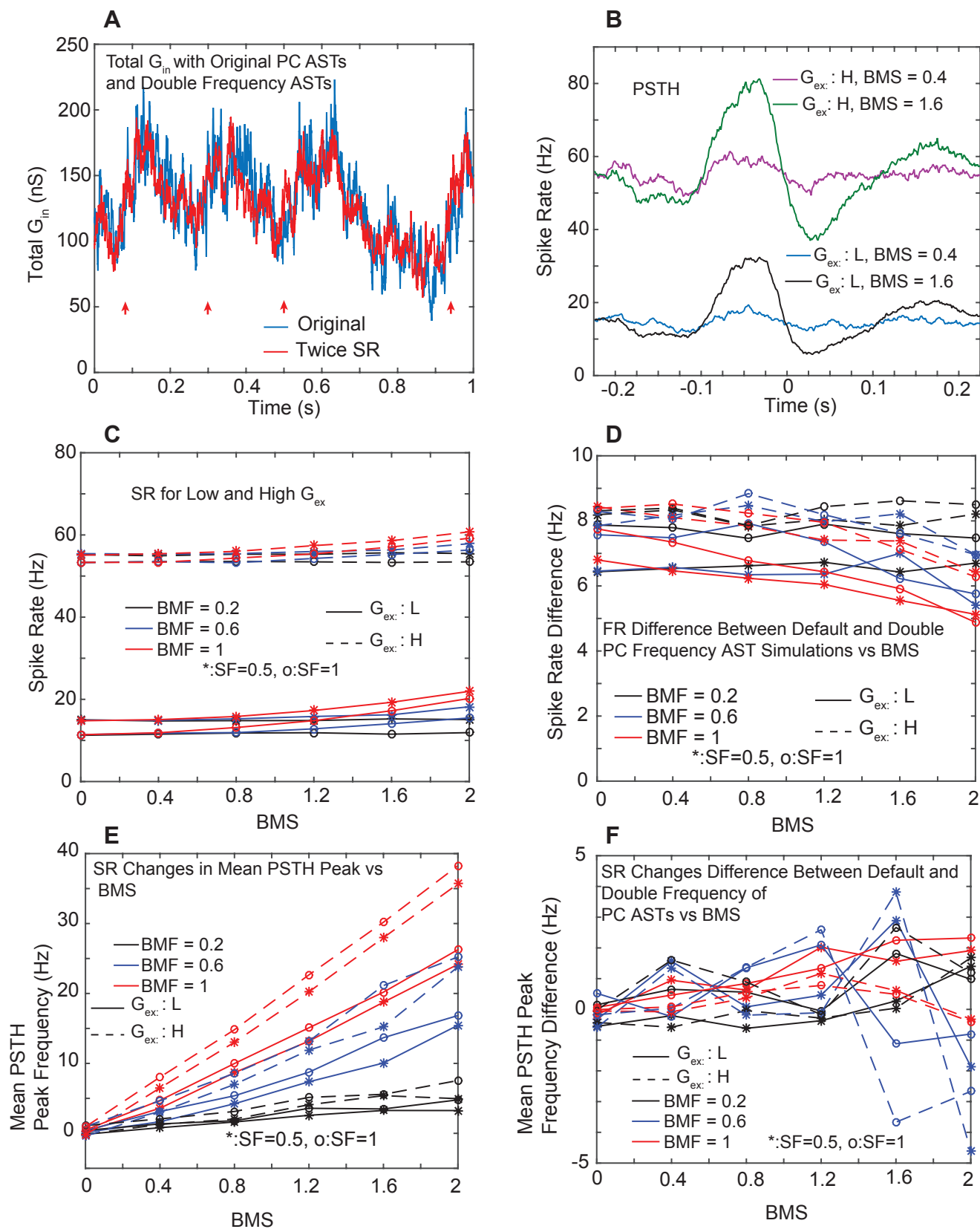

Supplement: S6 Fig — The Gin was adjusted from 16 to 9.84 nS to result in the same level of total inhibition as for the default PC spike rate. The adjustment was less than half because the steady state depression level at double the firing rate was increased. Panel annotation and methods used are as in Figs 6–8 and S5. (PDF) [file pcbi.1005578.s007.pdf]

Supplemental Figure 7

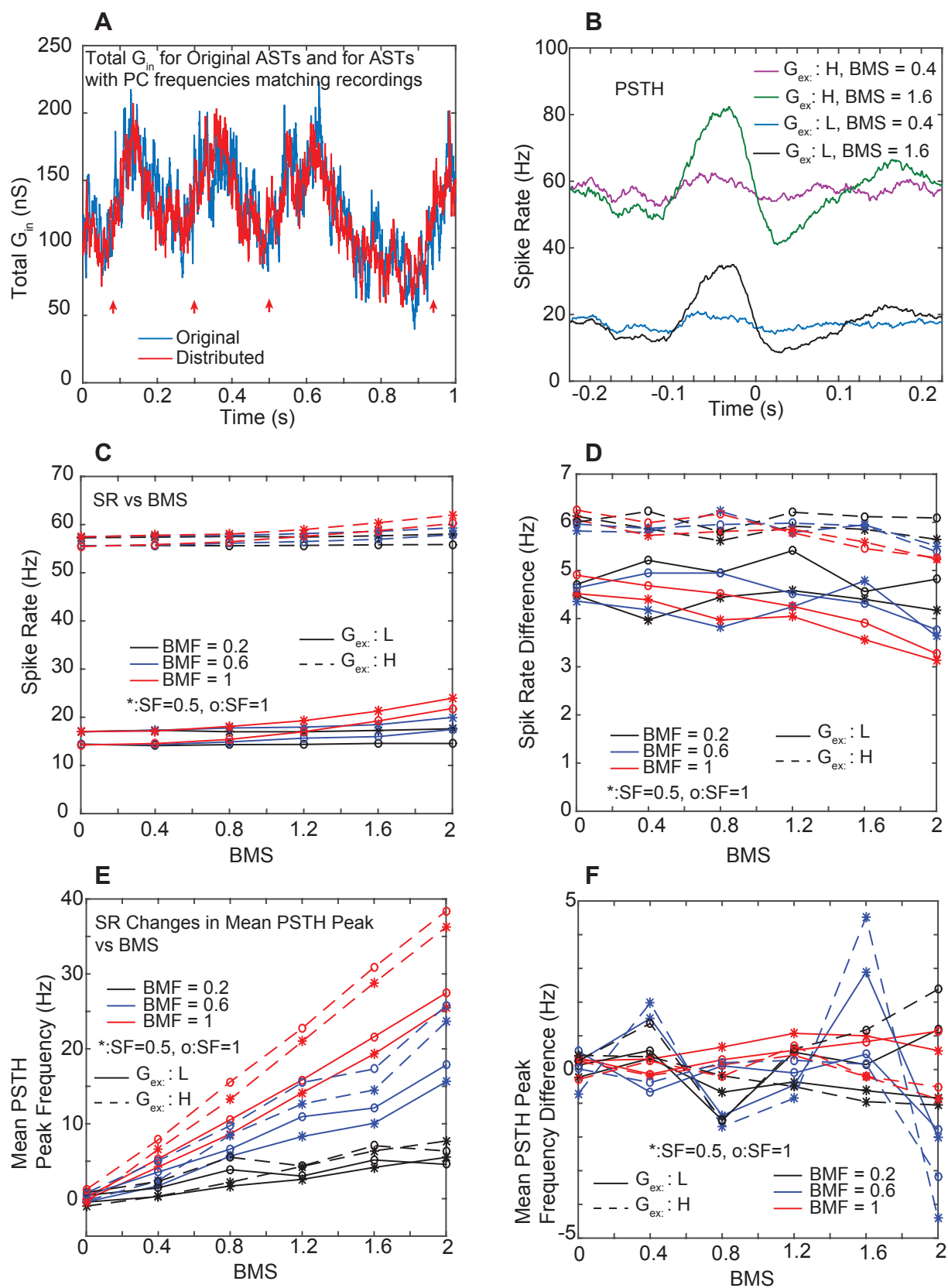

Supplement: S7 Fig — The Gin was set to 12.37nS for this set, as this resulted in a good match for the total inhibitory input conductance. Different PC spike rates for ASTs were obtained from the same rate template by scaling the normalized template to a distribution of rates across ASTs matching the recorded rate distribution. This gives a higher weight to faster spiking inputs than lower spiking ones, which is partly offset by the different levels of steady state depression, however. Panel annotation and methods used are as in Figs 6–8 and S5. (PDF) [file pcbi.1005578.s008.pdf]

Supplemental Figure 8

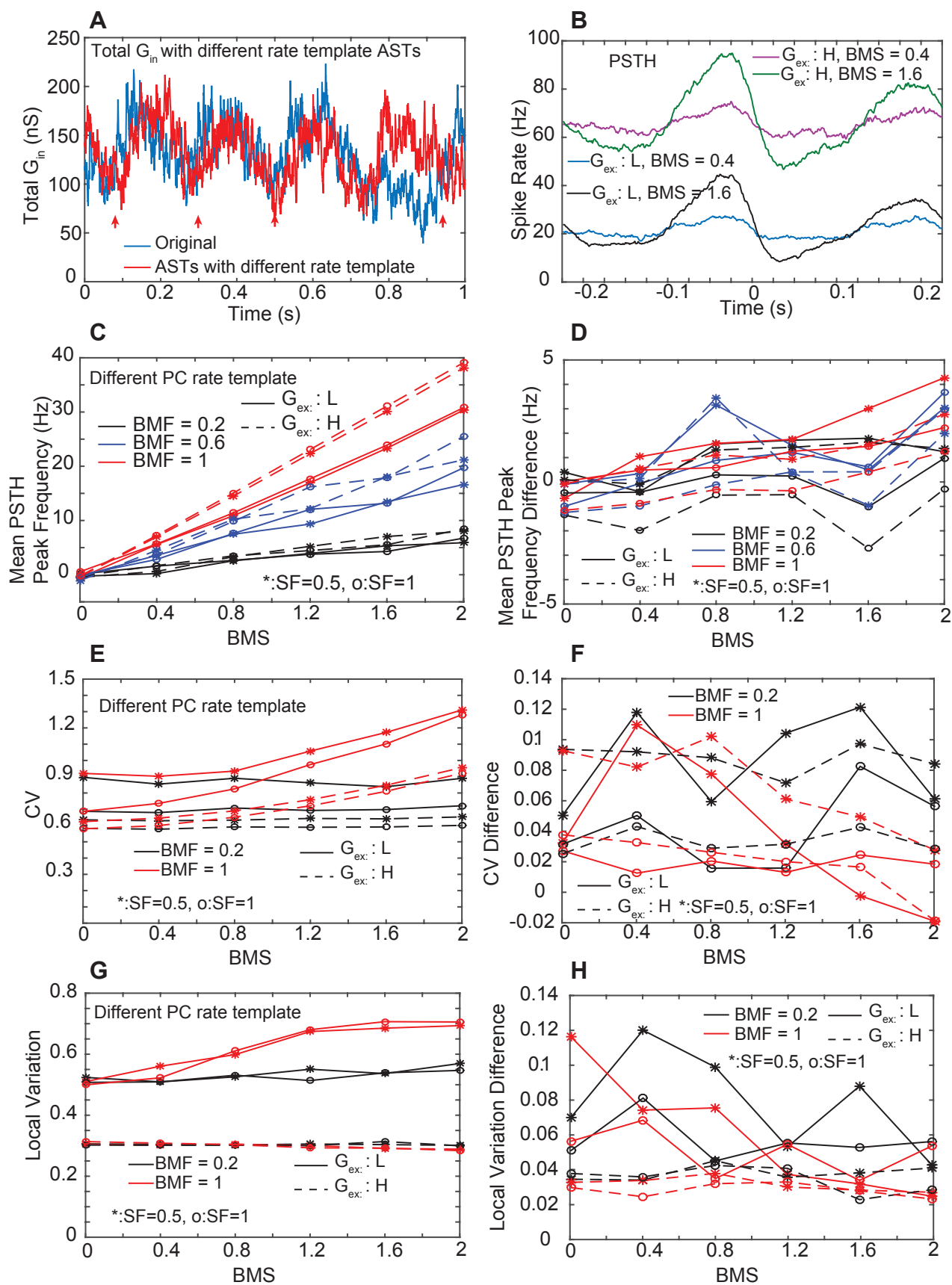

Supplement: S8 Fig — The PC with the new rate template had a spike rate of 59.9 Hz, a lower CV of 0.43 (compared to CV = 0.67 for the default), and a lower LV of 0.18 compared to LV = 0.31 for default). Panel annotation and methods used are as in Figs 6–8, S4 and S5. (PDF) [file pcbi.1005578.s009.pdf]

Supplemental Figure 9

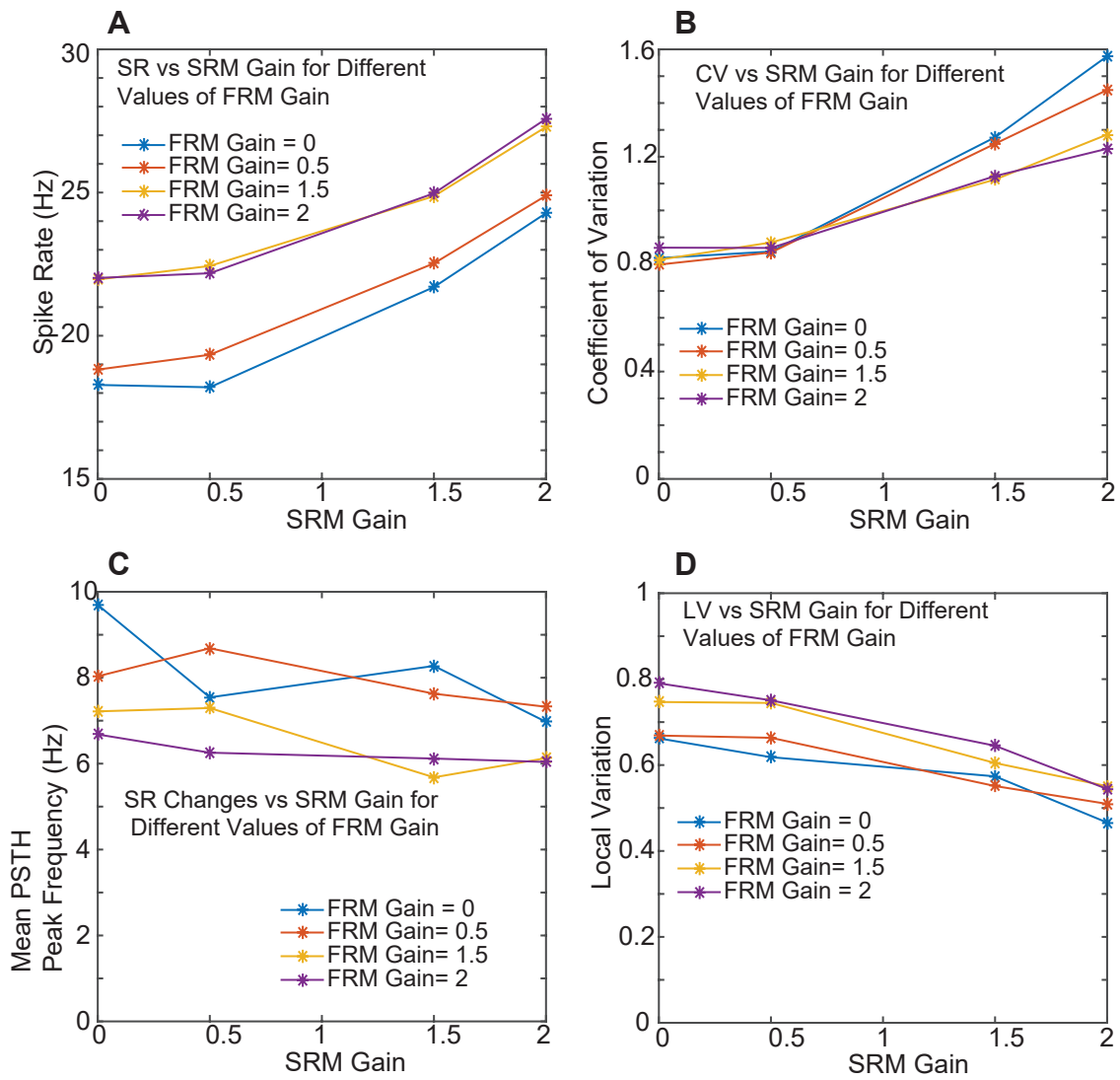

Supplement: S9 Fig — Gex = 3.5 nS, Gin = 16 nS, BMS = 0.8, BMF = 0.8 for all panels. (PDF) [file pcbi.1005578.s010.pdf]
